# Tracing groundwater circulation in a valuable mineral water basin with geochemical and isotopic tools: the case of *FERRARELLE*, Riardo basin, Southern Italy

**DOI:** 10.1007/s10653-021-00845-x

**Published:** 2021-03-01

**Authors:** Elisa Sacchi, Emilio Cuoco, Harald Oster, Vittorio Paolucci, Dario Tedesco, Stefano Viaroli

**Affiliations:** 1 Department of Earth and Environmental Sciences, University of Pavia, Via Ferrata 9, Pavia, Italy; 2 Department of Environmental, Biological and Pharmaceutical Sciences and Technologies, University of Campania “L. Vanvitelli”, Via Vivaldi 43, 81100 Caserta, Italy; 3 Spurenstofflabor, Wachenheim, Germany; 4 Ferrarelle S.P.A., Contrada Ferrarelle, Riardo, Italy; 5 Sciences Department, University of Roma Tre, Largo S. L, Murialdo 1, 00145 Rome, Italy; 6 Istituto Nazionale di Geofisica e Vulcanologia, Osservatorio Vesuviano, Via Diocleziano 328, 80124 Napoli, Italy

**Keywords:** Stable isotopes, Aquifer recharge, Hydrogeological circuits, Groundwater dating, Fractured aquifers

## Abstract

The Riardo basin hosts groundwater exploited for the production of high quality, naturally sparkling, bottled water (e.g., Ferrarelle water), and circulating in a system constituted by highly fractured Mesozoic carbonates, overlain by more impervious volcanic rocks of the Roccamonfina complex. The two formations are locally in hydraulic connection and dislocated by deep-rooted faults. The study aimed at elucidating groundwater origin and circulation, using isotopic tracers (δ^18^O, δ^2^H, δ^11^B and ^87^Sr/^86^Sr) coupled to groundwater dating (Tritium, CFCs and SF_6_). Besides recharge by local precipitation over the Riardo hydrogeological basin, stable isotope ratios in water indicated an extra-basin recharge, likely from the elevated surrounding carbonate reliefs (e.g., Maggiore and Matese Mts.). The mineralization process, promoted by the deep CO_2_ flux, controls the B and Sr contents. However, their isotopic ratios did not allow discriminating between circulation in the volcanic and in the carbonate aquifers, as in the latter the isotopic composition differed from the original marine signature. Groundwater model ages ranged from ~ 30 years for the volcanic endmember to > 70 years for the deep, mineralized end-member, with longer circuits recharged at higher elevations. Overall, the results of this study were particularly relevant for mineral water exploitation. A recharge from outside the hydrogeological basin could be evidenced, especially for the more mineralized and valuable groundwater, and an active recent recharge was detected for the whole Riardo system. Both findings will contribute to the refinement of the hydrogeological model and water budget, and to a sustainable development of the resource.

**Supplementary information:**

The online version contains supplementary material available at 10.1007/s10653-021-00845-x.

## Introduction

Elucidating groundwater circulation in many aquifers is a difficult task, being the resource hidden (Daly 2009). This is a particularly relevant issue in complex hydrogeological systems, where mixing occurs between different waters (Baiocchi et al. 2013; Carrillo-Rivera 2000).

At a catchment scale, fractured aquifers may be regarded as continuous systems with discharge points corresponding to the springs, although it is often not possible to precisely identify a water table level that can be followed over long distances. Nevertheless, at the local scale, the fracture distribution and orientation become highly relevant for the development, and detailed structural studies are required to increase the chances of success (Celico et al. 2006; Petrella et al. 2015).

Carbonate aquifers represent highly relevant water reservoirs worldwide, providing large volumes of high-quality drinking water (Chen et al. 2017; Goldscheider et al. 2020). Volcanic aquifers are also important reservoirs in volcanic islands (Herrera and Custodio 2008; Izquierdo 2014) and in the Tyrrhenian side of Central Italy (Capelli et al. 2005; Manca et al. 2017; Piscopo et al. 2008, 2018). In Italy, approximately 2 million inhabitants nowadays depend on volcanic water for drinking (Baiocchi et al. 2011). However, the water quality is often affected by the presence of undesired natural contaminations, in particular, fluoride, arsenic and trace metals (Angelone et al. 2009; Cuoco et al. 2015; Preziosi et al. 2010; Vivona et al. 2007).

In mineral water basins, the accurate management of the groundwater resource is vital to avoid aquifer overexploitation and changes in the chemistry of the tapped water. The definition of a reliable hydrogeological conceptual model is therefore necessary, focusing on the recharge areas definition, on the characteristics of groundwater circulation and on the groundwater “age” (Enemark et al. 2019; Tóth 1970).

Geochemical and isotopic tracers have been successfully applied in groundwater investigation (Clark and Fritz 1997). Stable isotope ratios in water are ideal conservative tracers of groundwater circulation. If the isotopic fractionation with elevation in precipitation from the area has been assessed, they can be used to identify the mean recharge elevation of the tapped groundwater (Gat 1980). The chemical reactions controlling the mineral water composition and the water–rock interaction processes are studied using hydrochemistry (major and trace elements) and geochemical modelling.

To discriminate between different aquifers, boron (δ^11^B) and strontium (^87^Sr/^86^Sr) isotopes may be useful, if the source rocks have a distinct composition, providing that they behave as conservative tracers (Battistel et al. 2016; Liotta et al. 2017; Pennisi et al. 2000). Significant deviations from the conservative behavior may occur in presence of clays, that could alter both the B concentration and its isotopic composition through adsorption/desorption processes (Gonfiantini and Pennisi 2006; Palmucci and Rusi 2014, Palmucci et al. 2016) and increase the Sr isotope ratio by release of radiogenic ^87^Sr derived from the ^87^Rb decay (Blum and Erel 2005; Shand et al. 2009; Woods et al. 2000).

Finally, groundwater dating using Tritium generally provides a qualitative indication on the presence of recent recharge. More recently, groundwater dating with anthropogenic gases (CFCs and SF_6_) has been developed. These tracers of anthropogenic origin may provide precise indication of the year of infiltration (Oster et al. 1996; Plummer et al. 2006).

The Riardo Plain hosts the Ferrarelle mineralized springs, exploited for bottling activity since the XIX century. The peculiar naturally sparkling character is due to the presence of an elevated gas content (98–99% pure CO_2_). The richness of dissolved species in well-balanced proportions and the slightly acidic, calcium bicarbonate composition impart a unique taste that allows listing this mineral water among the more valuable in Europe. To preserve this resource, several hydrogeological, geochemical, and isotopic monitoring activities were carried out in the last decades to define the aquifer characteristics and hydrodynamics, and to identify the processes that determine the mineralization.

In this study, we investigated the processes occurring at the local scale (i.e., the area related to the mineralized springs of the Ferrarelle field), using geochemical and isotopic monitoring. However, to justify some of the observed patterns, we considered the whole hydrological basin as defined by Viaroli et al. (2018) (about 100 km^2^, corresponding to the Roccamonfina SE flank, part of the Riardo Plain and the NW sector of the Maggiore Mt.), together with some extra-basin waters from the surrounding carbonate reliefs, which could be representative of deeper and longer circuits. The main objectives were to identify the recharge areas, and to elucidate groundwater origin and circulation. Dating the main groundwater endmembers allowed revising the previous hydrogeological conceptual models (Cuoco et al. 2020; Viaroli et al. 2018). The results of this investigation and the information provided by the application of multiple tracers allowed defining the main features of the Ferrarelle mineral water basin for a long-term sustainable management of the resource.

### Study area

#### Geological setting

The study area, located in the northern Campania Region (Southern Italy) corresponds to the Roccamonfina Volcano, the Riardo Plain and the surrounding carbonate reliefs. In this sector, two main geological domains can be distinguished: the sedimentary units and the volcanic deposits mainly erupted from the Roccamonfina Volcano.

**Fig. 1:**
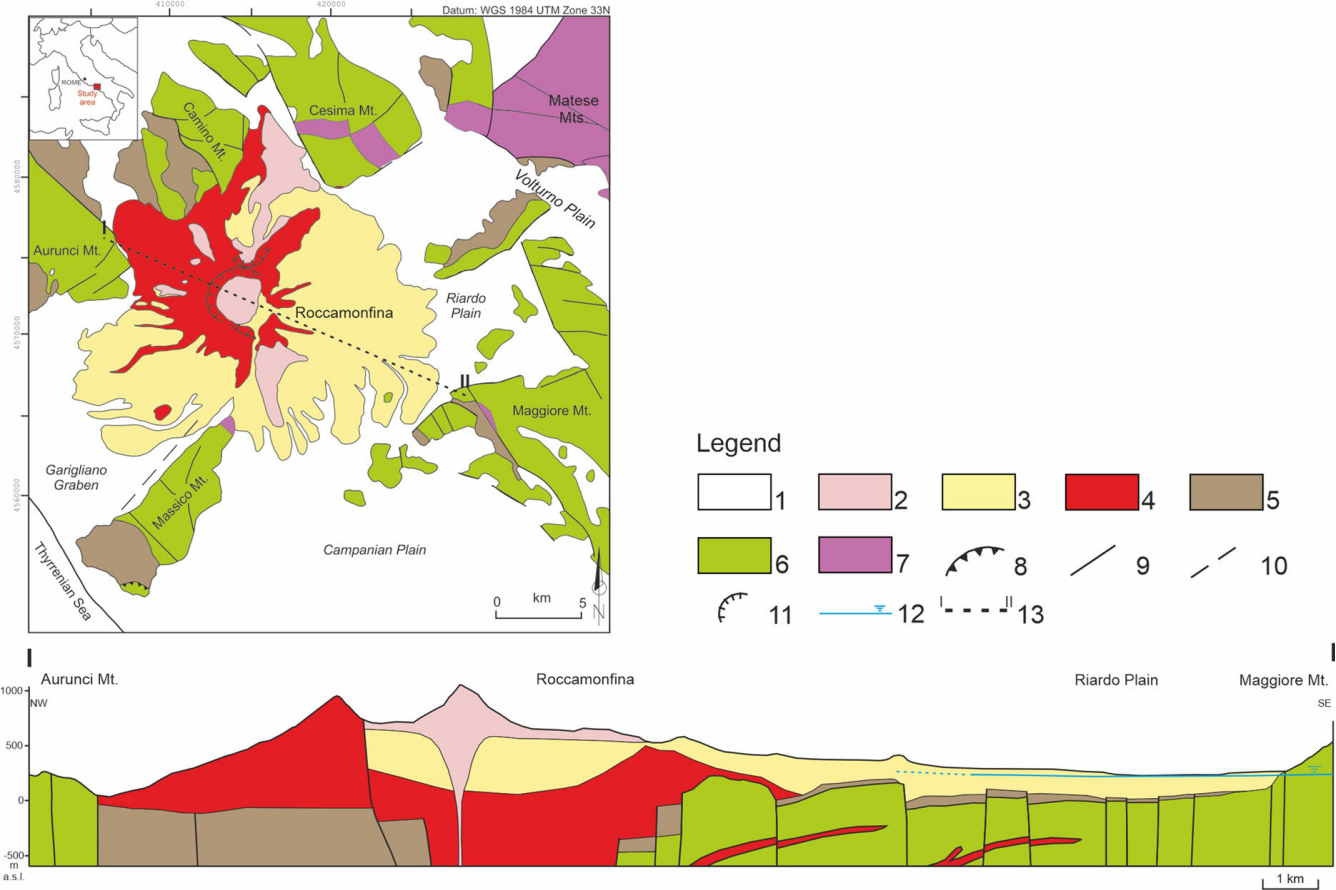
Schematic geological map of the study area (modified from Viaroli et al. 2019b). Legend: (1) Quaternary deposits; (2) Roccamonfina Volcano deposits, 3rd epoch; (3) Roccamonfina Volcano deposits, 2nd epoch; (4) Roccamonfina deposits, 1st epoch; (5) Flysch deposits; (6) Carbonate units; (7) Dolostone and dolomitic limestone units; (8) Thrust fault; (9) Normal and strike-slip faults involving the sedimentary basement; (10) Normal and strike-slip faults involving the quaternary units; (11) Roccamonfina caldera rim; (12) Basal groundwater level; (13) Geological cross-section trace

The sedimentary basement corresponds to the Meso-Cenozoic Apennine carbonate sequences, highly deformed since the Miocene during the orogenetic phase and later during the Plio-Pleistocene distensive tectonic activity, which displaced the basement via numerous horst and graben (Boncio et al. 2016; Cosentino et al. 2006; Giordano et al. 1995). Most of these structures are covered by the volcanic deposits, with the exception of the carbonate horst structure outcropping near the town of Teano, on the SE flank of the Roccamonfina Volcano. The carbonate units crop out in the Maggiore Mt., in the Matese Mts., and in other mountain ridges surrounding the Riardo Plain (Fig. 1). The oldest outcropping unit corresponds to the dolostone and dolomitic limestone unit (Trias) which is the base of the sedimentary sequence (D’Argenio and Pescatore 1962). The following deposited unit corresponds to dolomitic limestones and mainly limestones in shelf facies (Jurassic – Paleocene). After the involvement of this sector in the Apennine orogenesis, the carbonate sedimentation ended, and marl and synorogenic flysch units were deposited. According to the regional geologic reconstruction (Giordano et al. 1995; Saroli et al. 2014), the flysch deposits discontinuously outcrop in the study area and also underneath the Riardo Plain, suggesting that the flysch deposits should be preserved in the grabens and eroded on the horsts. The morphology of the sedimentary basement, under the volcanic deposits filling the plain, was defined in detail near the town of Riardo through the elaboration of data from tens of boreholes, drilled in the Ferrarelle mineral water area.

The Roccamonfina Volcano was active from around 550 to 150 ka (Rouchon et al. 2008) and its evolution can be divided into three epochs (De Rita and Giordano 1996). The first epoch consisted in the emplacement of the stratovolcano, by the deposition of mainly ultrapotassic lavas (HKS) and minor fall deposits. The second epoch was characterized by highly explosive activity and the deposition of ignimbrite units. The third epoch was characterized by an intense intra caldera volcanic activity with several phreatomagmatic eruptions and calcalkaline lava (KS) domes emplacement (De Rita and Giordano 1996) (Fig. 1).

The volcanic sequence filling the Riardo Plain could be summarized in four main units (Viaroli et al. 2019a). Listed from the older:

- Basal volcanoclastic unit (from ca. 440 to ca. 350 ka): made up by alternate reworked volcanic deposits.
- Brown Leucitic Tuff ignimbrite unit (ca. 350 ka): lithic tuff in ash matrix.
- Upper pyroclastic unit (from ca. 350 to ca. 330 ka): made up by alternation of reworked volcanic deposits and pumice–rich ignimbrite unit.
- Campanian Ignimbrite unit (39 ka): ignimbrite deposit in ashy facies erupted from the Campi Flegrei volcanic district.

#### Hydrogeological setting

The hydrogeological framework of the study area was investigated in several studies since the 1980s, both at regional (Boni et al. 1986; Celico 1978; De Vita et al. 2018; Viaroli et al. 2018) and at the local scale (Capelli et al. 1999; Mazza et al. 2013; Viaroli et al. 2016a, 2019b). Results agree defining the Roccamonfina Volcano hydrostructure and other carbonate aquifers. Roccamonfina Volcano is characterized by a radial drainage pattern of the basal aquifer of the volcano, moving toward the surrounding plains (Boni et al. 1986). The complexity of the volcano structure allows the formation of perched aquifers characterized by seasonal (Viaroli et al. 2016b) or perennial behavior. The most relevant perched aquifer was identified in the Roccamonfina caldera, where the local direct infiltration occurring over the upper portion of the volcano feeds a productive aquifer of approximately 150 L/s. However, the contribution of the caldera aquifer to the basal aquifer seems to be negligible (Viaroli et al. 2019b).

**Fig. 2:**
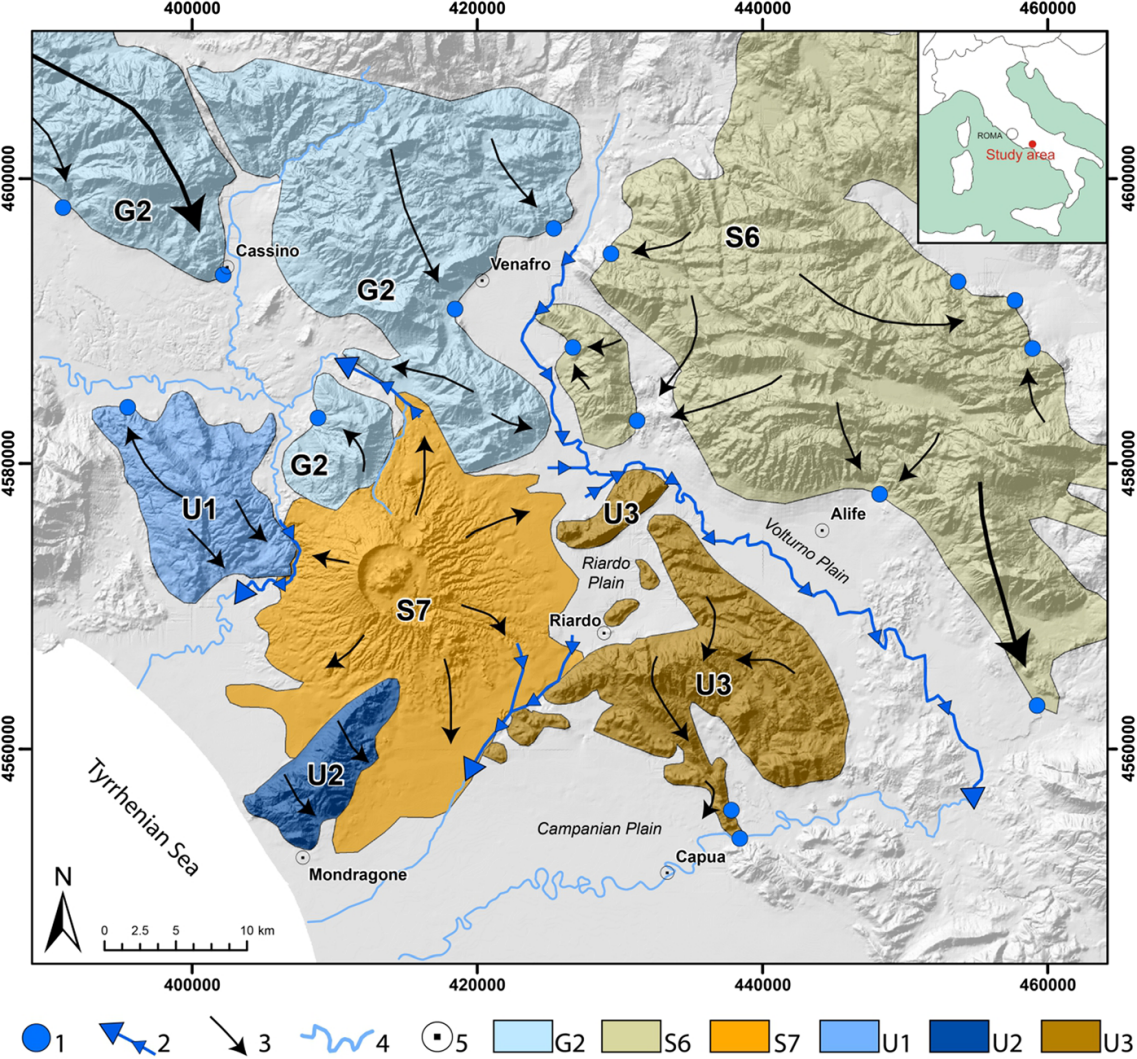
Map of the hydrostructures (modified after Viaroli et al. 2018). Legend: (1) Main springs (Q > 1 m^3^/s); (2) Gaining streams; (3) Groundwater discharge direction; (4) Main rivers; (5) Main cities. Hydrostructures: G2) M. Simbruini Mts., Ernici Mts., Cairo Mt., Camino Mt, Mainarde Mts. and Cesima Mt.; S6) Matese Mts. and Totila Mt.; S7) Roccamonfina Volcano; U1) Maio Mt.; U2) Massico Mt.; U3) Maggiore Mt

All carbonate aquifers (Fig. 2) feed very productive springs, discharging more than 1m^3^/s and located at the base of the carbonate structures, in correspondence to the boundary of the alluvial plains (Boni et al. 1986).

The relationships between the carbonate aquifers and the surrounding plains are not always clear, and still matter of debate. A lateral groundwater discharge from the Maggiore Mt. towards the Campanian Plain was recognized (Corniello et al. 1990, 2010) as well as between the Matese Mts. and the upper Volturno Plain (Rufino et al. 2020).

**Fig. 3:**
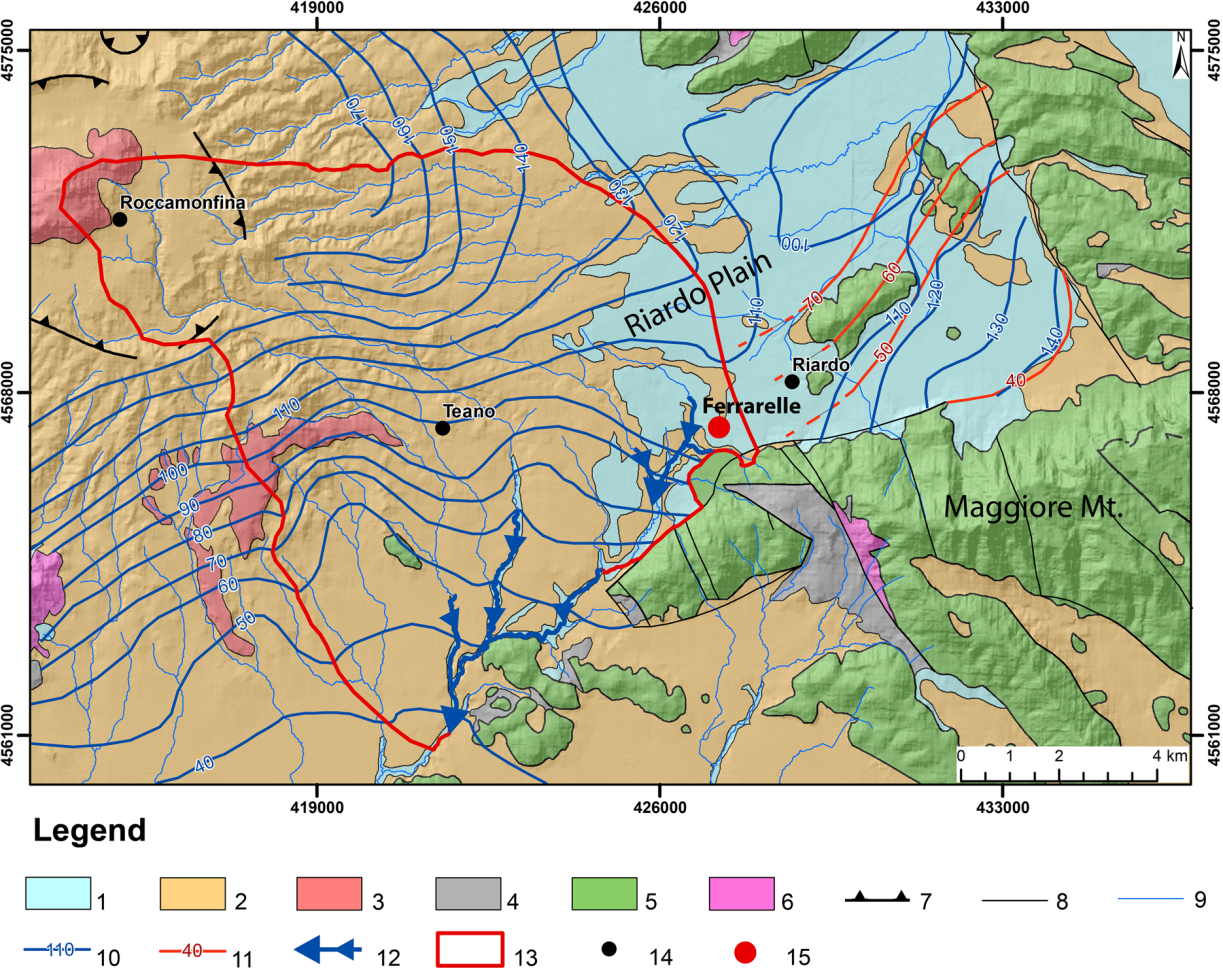
Hydrogeological map of Riardo Plain and Roccamonfina Volcano. Legend. (1) Alluvial deposits; (2) Pyroclastic deposits; (3) Lava; (4) Synorogenic flysch units; (5) Carbonate Units; (6) Dolomitic Units; (7) Caldera rim; (8) Main faults affecting the outcropping sedimentary units; (9) Hydrographic pattern; (10) Groundwater elevation map; (11) Groundwater elevation map of the confined carbonate aquifer; (12) Gaining streams; (13) Ferrarelle basin; (14) Main towns; (15) Ferrarelle bottling plant

In the Riardo Plain the hydrogeological framework is quite complex but two main areas can be identified (Fig. 3). In the eastern sector of the Riardo Plain, two aquifers can be distinguished: a deep confined carbonate aquifer and a shallow volcanoclastic aquifer, separated by a widespread layer of clay deposits (Viaroli et al. 2016a). The volcanoclastic aquifer is drained by gaining streams, with a mean NE discharge direction and hydraulic head ranging between 110 and 90 m a.s.l. On the contrary, the confined aquifer discharge is directed southward, with hydraulic head values decreasing from 70 to 40 m a.s.l. (Fig. 3).

In the western sector of the Riardo Plain, the absence of a widespread intermediate aquitard and the highly deformed basement allows the interconnection between the two aquifers. This results in a unique saturation level (approximately 110 m a.s.l.) given by the combination both volcanic and carbonate aquifers. On the contrary, the wavelength and frequency of head oscillations are only function of the volcanic aquifer behavior, excluding any detectable trend provided by the deep carbonate inflow (Viaroli et al. 2019a).

In this portion of the Riardo Plain, henceforth designated as Ferrarelle basin, previous studies (Cuoco et al. 2010, 2020) have identified the main hydrochemical types. Groundwater hosted in the volcanic aquifer has a low mineralization (Electrical Conductivity E.C. = 200–300 µS/cm), whereas groundwater with longer circuits in the carbonate aquifer acquires a higher mineralization (E.C. = 500–800 µS/cm), due to carbonate dissolution. These two groundwater types may be locally affected by inputs of deep CO_2_, strongly promoting water–rock interaction with host rocks and leading to a final highly mineralized water (E.C. = 2900–3100 µS/cm), showing an elevated bicarbonate, calcium and magnesium contents but also typical constituents of the volcanic rocks (e.g., Si, K, Na). According to Cuoco et al. (2020, 2010), the mixing between the lower mineralized volcanic endmember and the highly mineralized, CO_2_ saturated, carbonate endmember mostly occurs along the main faults, creating the Ferrarelle mineral springs. For this reason, groundwater mineralization is widely varying laterally (i.e., from one to the neighbouring well), as a function of the proximity to the conductive faults. Despite this heterogeneity, all the hydrochemical parameters vary continuously and linearly along the mixing line between the two main endmembers (Cuoco et al. 2020).

Although the local mixing phenomena occurring in the Ferrarelle basin were clearly defined (Cuoco et al. 2020), the recharge areas and the detailed groundwater discharge direction are still debated. Viaroli et al. (2018) highlighted a mismatching between the groundwater budget results over the hydrogeological basin defined on the groundwater divides of the volcanic aquifer and the hydrogeological monitoring data. The authors hypothesized a deep lateral inflow from the surrounding carbonate aquifers, and this hypothesis was numerically tested using a 2D mathematical model (Viaroli et al. 2019a). However, the recharge area of this additional groundwater contribution is still unknown.

The climate of the study area is Mediterranean with a dry and warm summer, and wet autumn and winter. The mean annual rainfall measured in the northern Campania Region is approximately 1000 mm, with a coefficient of variation of approximately 0.3 (Viaroli et al. 2018). The air temperature varies according to the ground elevation. In the study area the minimum monthly temperatures are recorded in January and February (approximately 6 °C) reaching approximately 26 °C in August.

### Materials and methods

**Fig. 4:**
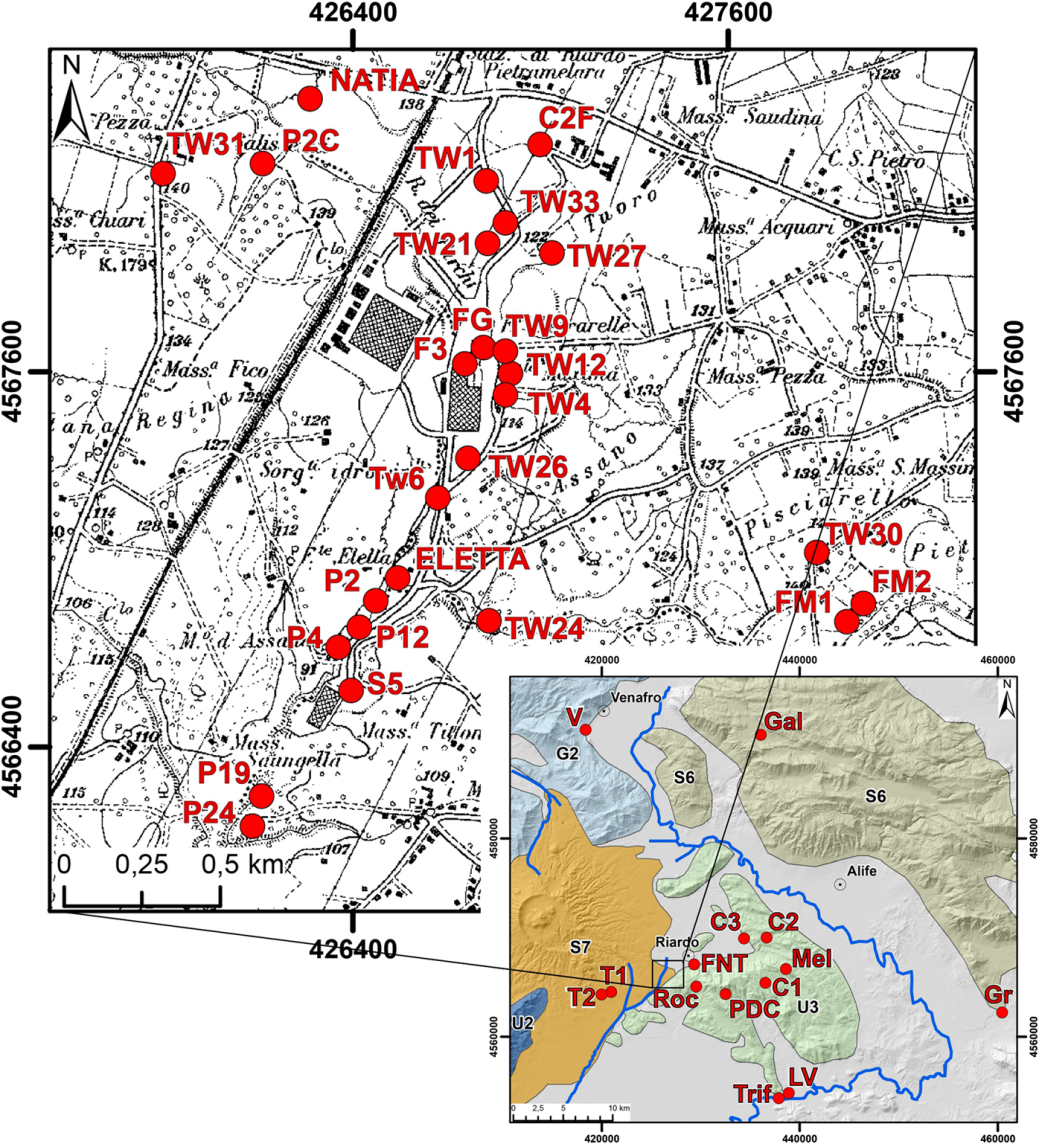
Location of all montioring and sampling points. Hydrostructures: G2) M. Simbruini Mts., Ernici Mts., Cairo Mt., Camino Mt, Mainarde Mts. and Cesima Mt.; S6) Matese Mts. and Totila Mt.; S7) Roccamonfina Volcano; U2) Massico Mt.; U3) Maggiore Mt

In this study, we present the results of several activities developed during the last decade. We considered groundwater samples collected in the Riardo Plain, both in the western sector (Ferrarelle basin) and in the eastern sector, where the carbonate aquifer and the overlying volcanic aquifer are separated. In addition, we collected groundwater from the Maggiore Mt. and Matese Mts. aquifers, in order to evaluate them as possible recharge areas (Fig. 4). The former was selected as it borders the Riardo Plain, while the Matese Mts. were investigated because characterized by different climate conditions.

Since 2010, samples from the Ferrarelle wells have been collected and analyzed for major ions and stable isotopes ratios in water twice per year, generally in May and in November. Similarly, starting from 2011, wells from adjacent areas and even outside of the hydrogeological basin were collected, and analyzed for the same parameters. To gain further insight on the mean recharge elevation of groundwater, two rainwater sampling stations were positioned on the Maggiore Mt., at an elevation of 525 m (P1) and 150 m (P2) a.s.l. Precipitation samples were preserved in tanks covered with vaseline oil to avoid any evaporation of the collected rain in the sampler, so as to be suitable for stable isotope analysis. In addition, six springs from the Maggiore Mt. area (Fontana di Monte—FNT, 150 m; Triflisco—Trif, 28 m; Rocchetta—Roc, 255 m; Pozzi di Croce—PDC, 525 m; Lago Verde—LV, 28 m and Melito—Mel 700 m a.s.l.), and three springs located in the Matese Mts. (Grassano—Gr, 55 m; Venafro—V, 177 m and Gallo Matese—Gal, 860 m a.s.l.) were also monitored.

Physico-chemical parameters, i.e., temperature (°C), pH and Electrical Conductivity (E.C. in µS/cm compensated at 20 °C) were measured during sampling with a multi-parameter probe (WTW 340i). Water samples were collected avoiding any external contamination and were filtered using a sterile 0.45 μm pore size cellulose acetate filter (Millipore™) before storing in prewashed PE bottles.

Major ions were analyzed at the Ferrarelle laboratory: alkalinity was measured by titration, and the other ions by chromatography (Dionex Dx120). The charge-balance error was generally below the recommended value of 5% (Clark 2015). The stable isotope ratios in water were determined by WS-CRDS (Wavelength-Scanned Cavity Ring Down Spectroscopy), using a Picarro INC L2120-i spectrophotometer at ISO4 (Turin, Italy). Results are expressed in δ‰ vs V-SMOW2 (Vienna-Standard Mean Ocean Water) and the analytical uncertainty (1σ) is ± 0.2 δ‰ for δ^18^O and ± 1 δ‰ for δ^2^H.

Based on the outcomes of previous work (Cuoco et al. 2020), samples belonging to the main hydrochemical facies and corresponding to circulations in the volcanic and in the carbonate aquifers were selected for the analysis of B and Sr isotopes. In addition, two rock samples obtained from the drill core of a Ferrarelle well (TW31), each representative of the volcanic and of the carbonate rocks of the Riardo basin, were also analyzed. Analyses were performed at ALS Scandinavia AB (Luleä, Sweden), by MC-ICP-MS after pre-concentration on ion exchange resins. Results are reported as δ^11^B‰ vs NBS-951 (analytical error ~ 0.8 δ ‰) and as absolute ratios for ^87^Sr/^86^Sr (analytical error ~ 0.00003).

Finally, productive wells were subject to groundwater dating using dissolved CFCs (CFC-11, CFC-12 and CFC-113) and SF_6_. To avoid any contamination by contact with the atmosphere, samples were collected in glass bottles filled below water, tapped and preserved in tightly sealed tin cans, also filled with the water sample. Gas concentrations were determined by headspace gas extraction (SF_6_) and by purge and trap technique (CFCs) followed by chromatographic analysis, as reported in Plummer et al. (2006), at Spurenstofflabor (Wachenheim, Germany). The analytical method is very sensitive and the uncertainty is reported in the table of results. For comparison, Tritium measurements were also performed, analyzed by liquid scintillation counting after electrolytic enrichment at Hydroisotop GmbH, Germany (method QMA 504–2/1: 2011–09). Results are expressed in Tritium Units (T.U.), together with the analytical uncertainty (double standard deviation), and are related to date of measurement (no half-life correction).

## Results

### Major ions

The results of the hydrochemical analyses are reported in Table 1.

**Table 1:** Groundwater hydrochemical composition

ID	Date	T °C	pH	E.C. µS/cm	HCO_3_^−^mg/L	F^−^mg/L	Cl^−^mg/L	NO_3_^−^mg/L	SO_4_^2−^ mg/L	Na^+^mg/L	K^+^mg/L	Mg^2+^ mg/L	Ca^2+^ mg/L
Eletta	04/06/2013	nd	5.9	1688	*1145*	1.1	18	9.6	3.6	50	47	26	380
F3	10/05/2012	15.5	6.0	1495	*1277*	0.6	16	7.8	2.8	51	48	18	334
FG	22/09/2014	16.0	6.0	1662	1354	1.0	15.6	4.4	5.8	48	46	20.0	379
Natia	09/05/2012	14.5	6.4	355	*193*	0.8	14	12	4.1	29	29	4.6	30
Natia	05/06/2013	nd	6.5	350	177	1.2	14.8	11.6	5	29	27	5.1	30
Natia1	22/09/2014	15.0	6.5	374	214	1.0	14	9.4	3.8	29.7	27.1	5	36
Natia2	22/09/2014	15.0	6.2	402	268	1.4	14.1	12	4	29	25	5	56
P12	14/10/2015	15.5	6.1	1227	921	1.2	16.6	5.5	5.5	42.3	31.6	17.8	243
P19	09/05/2012	16.0	6.0	1430	1121	1.0	19	0.1	5.0	55	41	20	280
P24	13/05/2013	18.5	6.3	3080	2745	1.2	20	0.1	1.2	80	80	48	640
P4	14/10/2015	15.5	6.1	1439	1110	1.4	16.2	8.1	8.1	63	45.4	21	277
TW1	13/05/2013	15.5	6.5	1000	738	1.5	14	6.7	5.5	30	30	8.2	199
TW1	04/06/2013	nd	6.3	880	744	1.4	14.6	7.3	5.5	34	32	8.7	191
TW12	23/09/2014	15.0	6.2	703	518.5	1.1	13	10.6	3.3	31	24	6.7	135
TW21	22/09/2014	16.0	6.5	955	707.6	1.5	14.1	7.3	4.4	32	25	8	190
TW24	23/05/2012	nd	6.4	3000	2588	1.3	22.2	< 0,7	1.4	85	82	49	663
TW24	13/05/2013	17.5	6.3	3120	2782	1.1	20	0.1	1.2	80	82	50	696
TW26	14/10/2015	16.5	6.8	948	695	1.5	13.2	7.8	3.8	31.8	28.4	9	187
TW27	09/05/2012	15.5	6.7	935	*694*	1.1	14	8.5	4.7	30	26	8.6	188
TW27	14/10/2015	15.5	6.6	1130	842	1.4	14.5	5.6	7.2	37.7	33.1	9.1	234
TW30	15/05/2013	16.0	6.2	2350	1970	1.2	19	2.3	2.9	61	47	40	532
TW30	04/06/2013	nd	6.0	2290	1964	1.5	18.7	2	3.4	58	45	43	572
TW31	10/05/2012	17.0	6.4	2995	*2826*	0.7	26	0.1	2.4	86	90	81	690
TW31	22/09/2014	17.0	6.2	3050	2812	0.9	32.7	2.1	4.7	79.3	71.6	58.5	693
TW4	10/05/2012	15.0	6.6	715	503	0.7	14	11	3.7	30	25	7.9	127
TW4	23/09/2014	15.0	6.2	710	518.5	1.0	13.6	10.6	3.3	31	25	7.0	137
TW6	23/05/2012	nd	6.1	1980	1494	1.5	21	0.1	2.8	65	65	29.0	420
TW9	23/09/2014	15.0	6.2	735	542.9	1.1	13.6	10.4	3.5	34	29	8	134
FM1^a^	14/05/2012	14.5	7.4	388	*337*	0.05	15	1.5	8.5	8.5	1.2	17	87
FM1^a^	22/05/2012	nd	7.4	388	232	0.1	15	1.39	8.4	8.2	1.22	17	88
FM2^a^	14/05/2012	15.0	7.4	424	*289*	0.05	12.6	6.6	6.7	9.8	2.7	13	75
C1^a^	25/03/2016	nd	7.0	455	360	0.3	11.4	5.8	5	7.6	4	17	71
C2^a^	07/04/2016	nd	6.9	781	573	0.4	13.3	7	5.7	12.4	7.9	15.3	154
C3^b^	30/04/2016	nd	6.7	951	744	1.0	14.1	14.6	7.3	22.6	18.7	46	146
T1^c^	28/11/2016	nd	7.1	765	640.5	1.4	18.8	10.6	7.8	26	26	15.7	137
T2^c^	28/11/2016	nd	7.0	676	610	1.2	19	13.4	7.2	29	26	12	113

Bicarbonate values in italics were calculated based on ionic balance

nd = not determined

^a^Wells tapping the Maggiore Mt. aquifer

^b^Well from the Riardo Plain

^c^Wells from the Teano carbonate horst

Groundwater shows widely varying mineralization, ranging in E.C. from 350 to 3120 µS/cm. Lower mineralization is detected in waters circulating within the volcanic aquifer only (Natia type) or within the carbonate aquifer of the Maggiore Mt., whereas samples with higher mineralization belong to the Ferrarelle basin. The dominant anion is bicarbonate, ranging from 177 to more than 2800 mg/L, while the other anions show a much lower variability (Cl^−^ from 11.4 to 32.7; NO_3_^−^ from 0.1 to 14.6; SO_4_^2−^ from 1.2 to 8.5 mg/L). Among cations, Ca^2+^ shows the highest and more variable concentrations, ranging from 30 to 696 mg/L. The other cations display similar concentrations and variability: Na^+^ from 7.6 to 86, K^+^ from 1.2 to 90 and Mg^2+^ from 4.6 to 81 mg/L.

### Stable isotope ratios in water

Rainwater was collected at two monitoring stations located on the Maggiore Mt., at an elevation of 525 m (P1) and 150 m (P2) a.s.l., from March 2014 to October 2016, in the form of cumulative samples. Results are reported in Table S1 in the Supplementary Material, together with the precipitation amount. The calculated amount-weighted average is reported in Table 2. The table also reports the amount-weighted average measured in the 1990s in three other stations located in the Riardo basin (Riardo, 110 m a.s.l.; Campagnola, 350 m a.s.l.; Roccamonfina, 620 m a.s.l.) (Longinelli 1994; Longinelli and Selmo 2003). It should be noted that there is no clear relationship between the isotopic composition of precipitation and the elevation of the sampling station. The higher delta values are recorded at the Riardo station, whereas the lower are found at P1, located at a lower elevation than the Roccamonfina station. However, this absence of relationship could also be due to the difference in sampling periods and duration.

**Table 2:** Stable isotope ratios in rainwater

Station	Elevation (m a.s.l.)	δ^18^O ‰ vs V-SMOW2	δ^2^H‰ vs V-SMOW2	Sampling period
P1	525	− 7.14	− 42.4	03/2014–04/2016
P2	150	− 6.40	− 38.6	03/2014–09/2016
Riardo^a^	150	− 6.03	− 35.9	01/1992–12/1998
Campagnola^b^	350	− 6.33	− 37.4	03/1992–11/1993
Roccamonfina^a^	620	− 6.64	− 37.8	03/1992–10/2000

^a^Data from Longinelli and Selmo (2003)

^b^Data from Longinelli (1994)

Spring monitoring data are reported in Table S2 in Supplementary Material (individual measurements) and summarized as average values in Table 3. Even in this case, there is no clear relationship with the elevation of the spring. The recharge elevation estimation is usually very difficult to assess, especially in case of karst springs where the effect of snow melting and the structural framework could increase the uncertainties on the groundwater flow (Lucianetti et al. 2020).

**Table 3:** Stable isotope ratios in spring water, and number of samples taken in the sampling period

Station	ID	Elevation (m a.s.l.)	δ^18^O ‰ vs V-SMOW2	δ^2^H‰ vs V-SMOW2	Samples	Sampling period
Fontana di Monte	FNT	150	− 6.19	− 33.2	13	2014–2020
Melito	Mel	700	− 6.71	− 35.8	3	2014–2015
Triflisco	Trif	28	− 6.63	− 37.4	14	2018–2020
Rocchetta	Roc	255	− 6.22	− 34.1	8	2018–2019
Pozzi di Croce	PDC	525	− 6.30	− 34.5	3	2019
Lago Verde	LV	28	− 6.60	− 37.1	8	2019–2020
Grassano	Gr	55	− 7.92	− 46.4	14	2011–2019
Venafro	V	177	− 7.33	− 42.6	14	2011–2019
Gallo Matese	Gal	860	− 8.31	− 48.6	14	2011–2019

In addition to springs, an extensive well sampling campaign was conducted in 2010 in the Ferrarelle basin. Following the results, six productive wells were selected and monitored about twice per year, in the period 2010–2019. Detailed results are reported in Tab. S3 and in Fig. S1 in Supplementary Material, and summarized as average values in Table 4. As observed in most hydrogeological environments, the isotopic ratios in groundwater vary less than in rainwater. In our case, the δ ^18^O values range between − 6.92 and − 6.02‰, i.e., less than 1‰ difference, despite the widely varying mineralization degree (see Table 1).

**Table 4:** Stable isotope ratios in groundwater, average values of samples taken in the sampling period

Well	δ^18^O ‰ vs V-SMOW2	Standard deviation	δ^2^H‰ vs V-SMOW2	Standard deviation	Sampling period
C1F	− 6.08	0.13	− 36.6	0.0	2010
Eletta	− 6.35	0.08	− 37.7	0.8	2010
F3	− 6.41	0.06	− 37.6	1.1	2010
P12	− 6.25	0.08	− 38.1	2.3	2010
P19	− 6.56	0.00	− 38.8	1.8	2010
P2	− 6.56	0.00	− 38.8	1.8	2010
P24	− 6.89	0.12	− 38.1	1.3	2010–2019
P2C	− 6.12	0.16	− 36.6	0.5	2010
S5	− 6.76	0.09	− 37.4	1.1	2010–2019
TW1	− 6.04	0.10	− 36.1	0.7	2010
TW24	− 6.92	0.11	− 38.1	1.4	2010–2019
TW26	− 6.14	0.05	− 36.6	0.1	2010
TW30	− 6.52	0.17	− 36.3	1.0	2010–2019
TW31	− 6.87	0.13	− 37.6	0.4	2011–2019
TW4	− 6.20	0.00	− 36.3	1.3	2010
TW6	− 6.44	0.16	− 35.8	1.0	2010–2019
FM1^a^	− 6.02	0.15	− 32.9	0.8	2011–2013
FM2^a^	− 6.10	0.07	− 33.0	0.4	2011–2013

^a^Wells tapping the Maggiore Mt. aquifer

### Boron and strontium isotopes

The results of 17 groundwater samples examined in the study are reported in Table 5. Boron concentrations range between 15 and 1260 µg/L. The lowest concentrations (15–19 µg/L) are displayed by samples collected in the carbonate aquifer of the Maggiore Mt. Slightly higher concentrations characterise the volcanic endmember (99 µg/L). The highest concentrations are recorded in the mineralized waters of the Ferrarelle basin, and are likely derived from the enhanced water–rock interaction caused by the deep CO_2_ input (Cuoco et al. 2020). Indeed, B concentrations show a significant linear correlation with E.C. (*n* = 17; *R*^2^ = 0.965; *p* < 0.01).

**Table 5:** Boron concentrations and isotope composition of groundwater samples

ID	Date	Location	E.C. µS/cm	B µg/L	δ^11^B‰ vs NBS-951
FM1	14/05/2012	Maggiore Mt	388	15	20.9
FM2	14/05/2012	Maggiore Mt	424	19	9.5
Natia	09/05/2012	Ferrarelle basin	355	99	− 4.9
TW27	09/05/2012	Ferrarelle basin	935	150	− 6.3
TW4	10/05/2012	Ferrarelle basin	715	280	− 3.6
P19	09/05/2012	Ferrarelle basin	1430	425	− 8.0
F3	10/05/2012	Ferrarelle basin	1495	560	− 4.2
TW31	10/05/2012	Ferrarelle basin	2995	1260	− 5.0
TW24	13/05/2013	Ferrarelle basin	3120	1250	− 8.7
P24	13/05/2013	Ferrarelle basin	3080	1160	− 6.9
TW30	15/05/2013	Ferrarelle basin	2350	750	− 8.6
TW1	13/05/2013	Ferrarelle basin	1000	140	− 9.8
C1	25/03/2016	Maggiore Mt	455	35	− 1.4
C2	07/04/2016	Maggiore Mt	781	100	− 5.2
C3	30/04/2016	Riardo Plain	951	131	19.49
T1	28/11/2016	Teano	765	100	− 4.48
T2	28/11/2016	Teano	676	105	− 4.77

Boron isotope composition is also highly variable, from + 20.9 to − 9.83 δ^11^B‰. However, no linear correlation is observed with the B concentrations or with E.C.

The Sr isotope composition was measured on seven samples representing the groundwater endmembers, to test the suitability of this isotopic systematics to trace groundwater circuits. Results are reported in Table 6.

**Table 6:** Sr concentrations and isotopic composition of groundwater samples

ID	Date	Location	E.C. µS/cm	Sr µg/L	^87^Sr/^86^Sr
Natia1	22/09/2014	Ferrarelle basin	374	100	0.70890
TW31	22/09/2014	Ferrarelle basin	3050	1670	0.70895
C1	25/03/2016	Maggiore Mt	455	37	0.70849
C2	07/04/2016	Maggiore Mt	781	216	0.70820
C3	30/04/2016	Riardo Plain	951	345	0.70790
T1	28/11/2016	Teano	765	245	0.70916
T2	28/11/2016	Teano	676	274	0.70897

Sr concentrations range between 37 and 1670 µg/L. The lowest concentration (37 µg/L) corresponds to a sample from the carbonate aquifer of the Maggiore Mt. A slightly higher concentration characterises the volcanic endmember (100 µg/L). The highest concentration is recorded for the mineralized water of the Ferrarelle basin. Concentrations show a significant linear correlation with E.C. (*n* = 7; *R*^2^ = 0.992; *p* < 0.01) suggesting the same origin of B, from the dissolution of the aquifer matrix promoted by the deep CO_2_ input.

Strontium isotope ratios vary between 0.70790 and 0.70916 and do not correlate to E.C. or to the Sr content.

### Dissolved anthropogenic gases and Tritium

The available results of CFCs and SF_6_ measurements are reported in Table 7.

**Table 7:** Results of CFCs and SF_6_ measurements

ID	Date	CFC-12 (pmol/L)	CFC-11 (pmol/L)	CFC-113 (pmol/L)	SF_6_ (fmol/L)
FM1^a^	22/05/2012	1.9 ± 0.1	3.6 ± 0.4	0.30 ± 0.05	na
Natia	22/05/2012	5.1 ± 0.3	2.6 ± 0.3	0.04 ± 0.05	na
F3	23/05/2012	0.19 ± 0.05	0.16 ± 0.05	< 0.01	na
TW6	23/05/2012	0.11 ± 0.05	0.02 ± 0.05	< 0.01	na
TW24	23/05/2012	< 0.01	< 0.01	< 0.01	na
TW30	04/06/2013	0.02 ± 0.05	0.05 ± 0.05	< 0.01	0.2
TW1	04/06/2013	0.65 ± 0.05	0.36 ± 0.05	0.01 ± 0.01	4.5 ± 0.5
Eletta	04/06/2013	0.02 ± 0.05	10 ± 2	< 0.01	< 0.1
Natia	05/06/2013	5.5 ± 0.3	2.9 ± 0.3	0.04 ± 0.05	6 ± 1
TW12	23/09/2014	0.42 ± 0.05	0.20 ± 0.05	0.02 ± 0.05	5.9 ± 0.6
TW9	23/09/2014	0.45 ± 0.05	0.23 ± 0.05	0.02 ± 0.05	4.0 ± 0.4
TW4	23/09/2014	0.47 ± 0.05	0.23 ± 0.05	0.01 ± 0.05	4.9 ± 0.5
TW21	22/09/2014	0.53 ± 0.05	0.29 ± 0.05	0.01 ± 0.05	6.6 ± 0.7
FG	22/09/2014	0.06 ± 0.05	0.08 ± 0.05	< 0.01	< 0.1
Natia2	22/09/2014	6.7 ± 0.4	4.1 ± 0.5	0.07 ± 0.05	4.8 ± 0.5
TW26	14/10/2015	0.65 ± 0.05	0.33 ± 0.05	0.02 ± 0.01	6.5 ± 0.7
TW27	14/10/2015	0.68 ± 0.05	0.49 ± 0.05	< 0.01	7.2 ± 0.8
P4	14/10/2015	0.19 ± 0.05	3.3 ± 0.4	0.02 ± 0.05	0.2 ± 0.1
P12	14/10/2015	1.6 ± 0.1	2.9 ± 0.3	0.01 ± 0.05	0.1 ± 0.1

*na* not analyzed

^a^Well tapping the Maggiore Mt. aquifer

All the samples except TW24 show detectable amounts of CFCs and SF_6_, indicating that they contain post-1940 infiltrated water. The highest concentrations of CFCs are displayed by groundwater samples from both the Maggiore Mt. aquifer and the volcanic aquifer. Many samples from the Ferrarelle basin have a natural effervescence, and this may lead to the formation of little gas bubbles in the sampling bottle after collection. In this case, the relative amounts of the different gases could be influenced, depending on their solubility in water. This parameter decreases in the order CFC-11 > CFC-12 > CFC-113 > SF_6_, therefore SF_6_ could potentially be more affected and less reliable than CFC-11. Degassing could lead to inconsistencies in the results and possibly to an older age estimate. High concentrations of SF_6_ are recorded in most samples, regardless of their provenance. These concentrations are often exceeding the present day atmospheric level (i.e., samples show an SF_6_ excess).

Concerning Tritium, a large number of data are available from previous investigations conducted in the period 1992–1993 (Longinelli 1994), and only few measurements were made for comparison, as reported in Table 8.

**Table 8:** Results of the Tritium concentrations

Sample name	Date	Tritium (T.U.)	Average 1992–1993
FM1^a^	22/05/2012	3.2 ± 0.6	na
FM2^a^	22/05/2012	2.9 ± 0.5	na
Natia	22/05/2012	0.5 ± 0.5	2.3 ± 2
F3	23/05/2012	< 0,6	na
TW6	23/05/2012	< 0,6	0.8 ± 2
TW24	23/05/2012	< 0,6	na

Average in 1992–1993 from Longinelli (1994)

^a^Wells tapping the Maggiore Mt. aquifer

Tritium is present at detectable concentrations in groundwater from the Maggiore Mt. aquifer and from the volcanic aquifer, whereas it is absent in the mineralized samples from the Ferrarelle basin.

## Discussion

### Groundwater hydrochemistry

A detailed description of the groundwater hydrochemistry with a thoughtful discussion on the processes governing mineralization is reported in Cuoco et al. (2020). Therefore, in this work, hydrochemical data are used in comparison with those previously reported in order to ensure that the conclusions reached in our study can be generalized.

**Fig. 5:**
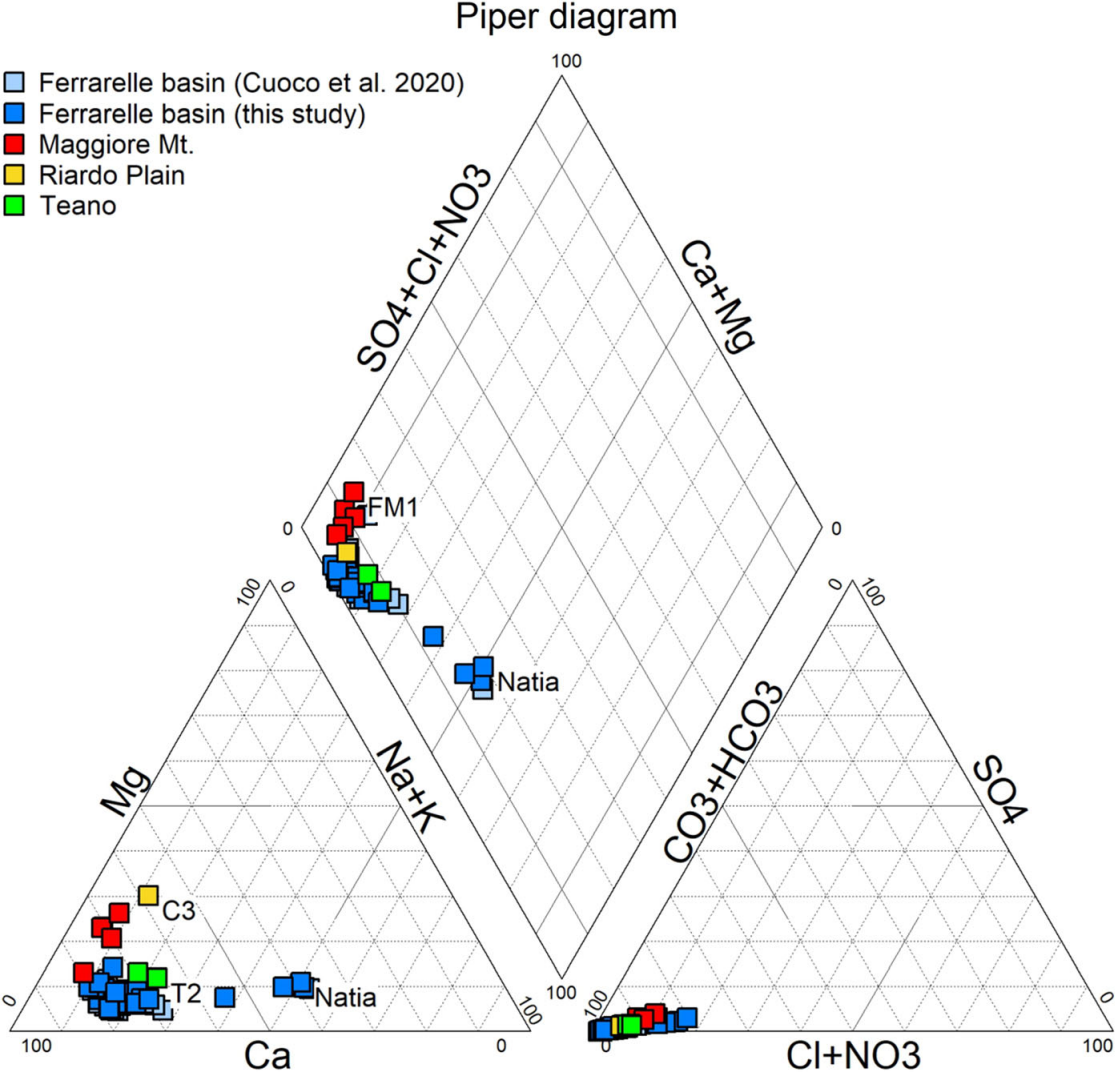
Piper diagram of the samples included in this study, compared to those reported by Cuoco et al. (2020)

The Piper diagram (Fig. 5) indicates that, in all samples, the dominant anion is HCO_3_^−^. Groundwater from the volcanic aquifer (e.g., Natia) shows higher percentages of Na^+^ + K^+^, whereas the rest of the samples are dominated by Ca^2+^. Only the sample from the Riardo Plain (C3) also shows a relatively higher Mg^2+^ content, likely derived from the dissolution of dolomitic limestones. Consequently, most of the samples are of Ca-HCO_3_ type or Ca-Na-HCO_3_ type, except for the Natia samples (Na-Ca-HCO_3_ type).

**Fig. 6:**
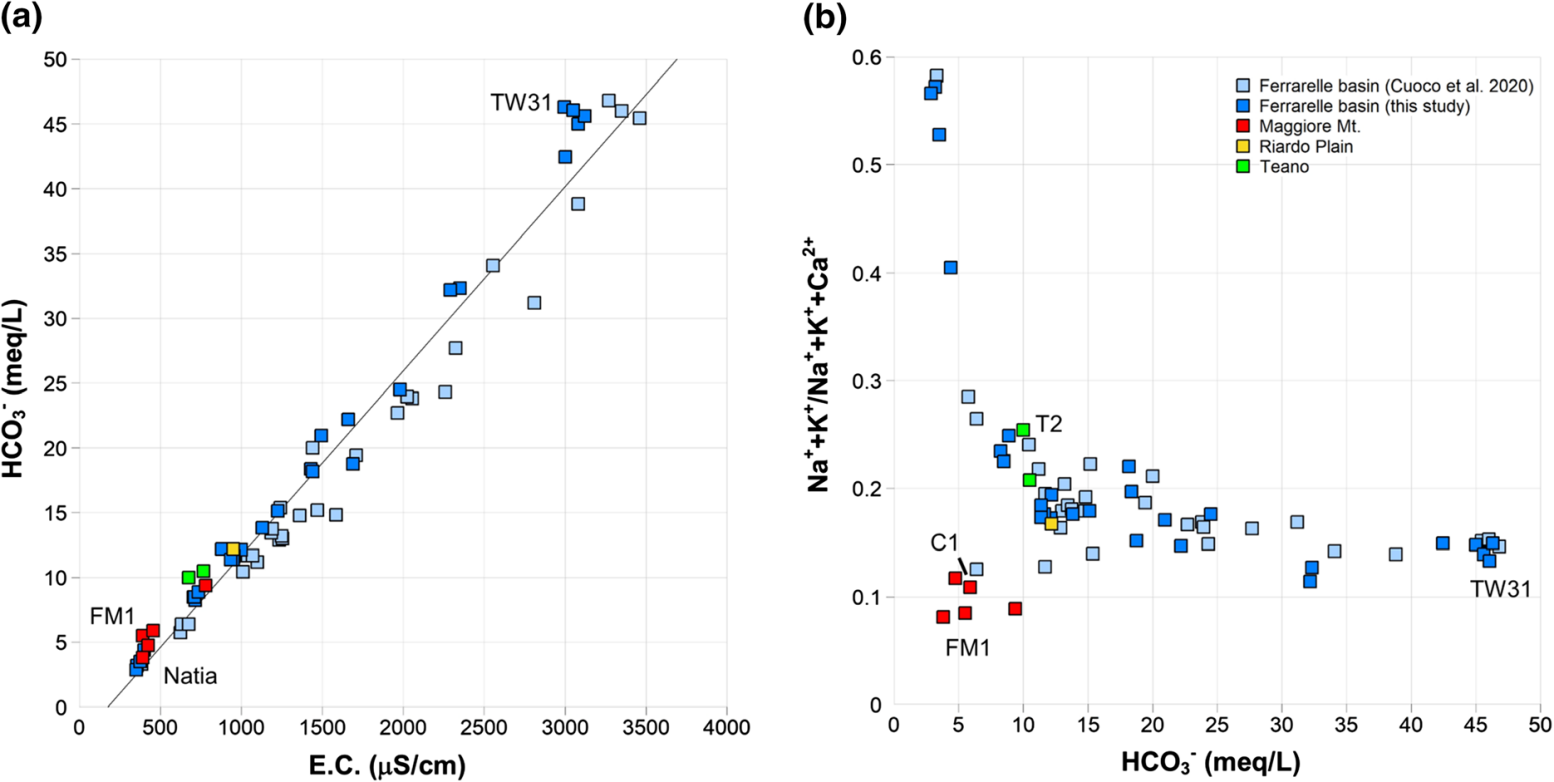
**a** Correlation between HCO_3_^−^ content and E.C.; **b** evolution of the relative proportion of Na^+^ + K^+^ (meq/L) with respect to Na^+^ + K^+^ + Ca^2+^ (meq/L) with increasing bicarbonate content

In the Ferrarelle basin, groundwater mineralization is strongly correlated to the bicarbonate content (Fig. 6a); therefore, E.C. can be used as a proxy for the degree of water–rock interaction. With increasing bicarbonate concentrations, the relative proportion of Na^+^ + K^+^ with respect to the sum of Na^+^ + K^+^ + Ca^2+^ tends to decrease (Fig. 6b). This finding indicates the shift from lower mineralized, Na^+^ + K^+^ dominated waters circulating in the volcanic aquifer to the highly mineralized Ca-HCO_3_ waters of the Ferrarelle basin. Only few samples from the Maggiore Mt. and from the Riardo Plain, circulating exclusively in carbonates, show lower relative proportions of Na^+^ + K^+^ and a low mineralization. The acquisition of a common cation ratio at increasing bicarbonate content (Fig. 6b) supports the indication that a contribution from the volcanic aquifer exists also in groundwater with the higher mineralization. These results are in perfect agreement with those of previous investigation (Cuoco et al. 2010, 2020), who found that carbonate hydrolysis increases in response to the deep CO_2_ input. However, in the Ferrarelle basin, the volcanic and the carbonate aquifers are not separated, and, even for high degrees of water–rock interaction, the contribution from the volcanic aquifer can be detected. In the discussion that follows, the E.C. values of the samples will be reported as an indication of the degree of water–rock interaction.

### Recharge areas

In earlier studies, the isotopic ratios displayed by the amount-weighted precipitation collected in the Riardo basin (Riardo, Campagnola and Roccamonfina stations) were combined with data from other monitoring stations to define the Southern Italy Meteoric Water Line SIMWL (Longinelli and Selmo 2003), with equation

$$ \delta^{2} H \, = \, 6.97 \, \delta^{18} O \, + \, 7.3165 $$

This meteoric water line is sensibly different from the Global Meteoric Water Line (Rozanski et al. 1993) for both slope and deuterium excess (*d*-excess ‰ = δ^2^H—8 δ^18^O; Dansgaard 1964), because of the Mediterranean influence (Gat and Carmi 1970).

**Fig. 7:**
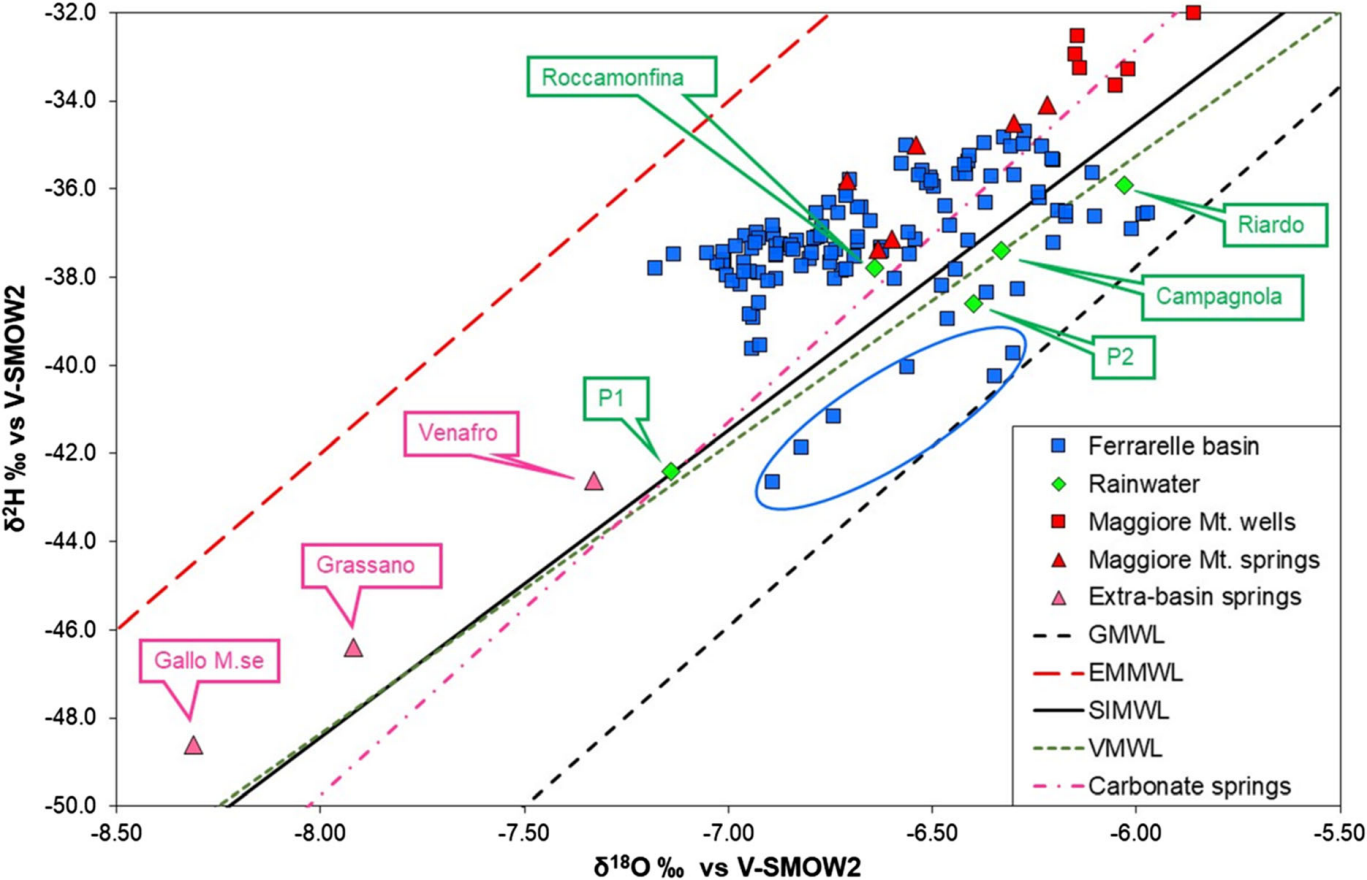
Isotopic composition of the Ferrarelle basin (blue squares) and of the Maggiore Mt. wells (red squares) and springs (red triangles), compared to amount-weighted precipitation (green diamonds), and to extra-basin springs (magenta triangles). GMWL = Global Meteoric Water Line (Rozanski et al. 1993); EMMWL = Eastern Mediterranean Meteoric Water Line (Gat and Carmi 1970); SIMWL = Southern Italy Meteoric Water Line (Longinelli and Selmo 2003); VMWL = Vulture Meteoric Water Line (Paternoster et al. 2008); regression line for carbonate springs from Central Italy (Minissale 2004)

In the plot δ^2^H vs δ^18^O (Fig. 7), Ferrarelle groundwater data fall close to the SIMWL and values are similar to those recorded in precipitation. Longinelli (1994) therefore concluded that the basin was recharged by local precipitation, with high δ^18^O values (> − 6.2‰) in water infiltrating in the middle-lower flank of the Roccamonfina Volcano (~ 180 m a.s.l.), and low δ^18^O values (< − 6.9‰) in the volcanic caldera (~ 630 m, but reaching the maximum elevation of 1005 m a.s.l.). Based on these isotopic results, the author stated that a recharge from the Maggiore Mt. was unlikely. However, the vertical isotope gradient in precipitation, of − 0.12‰ δ^18^O per 100 m elevation (Longinelli and Selmo 2003), was rather low, compared to the first gradient of − 0.18‰ reported in Longinelli (1994) and to other gradients reported in more recent literature (Giustini et al. 2016). For example, Paternoster et al. (2008) report a gradient value of − 0.17‰ for the Vulture Mt. and similarly, Petrella and Celico (2009) established a gradient of − 0.16‰ in the Matese Mts. Finally, recent hydrogeological investigation identified a perched aquifer in the Roccamonfina caldera, whose contribution to the basal aquifer and to the Riardo basin is considered negligible (Viaroli et al. 2019b).

**Fig. 8:**
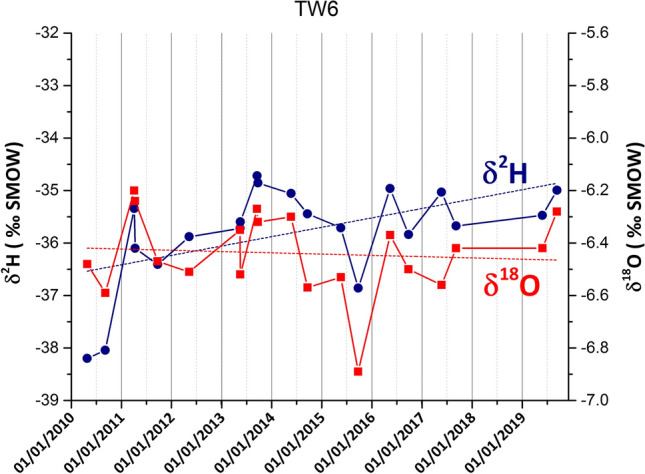
Evolution of the isotopic composition during the monitoring period. Dot lines highlight the isotopic trends. Data are reported in Tab. S3

In addition, most groundwater data plot above the SIMWL, except some samples evidenced with the blue oval in Fig. 7, which show a significantly lower δ^2^H. These data represent one set of monitoring data of the same productive wells and cannot be attributed to an analytical error. Indeed, examining the temporal trends of isotopic compositions for the Ferrarelle mineralized wells (Tab. S3 and Fig. S1), a progressive increase of δ^2^H can be noticed, not paralleled by a similar shift in the δ^18^O. An example of such trends is reported in Fig. 8. In other words, all the monitored wells are progressively increasing their *d*-excess. If an analytical error was involved (e.g., evaporation of the standards used in WS-CRDS), this would affect mostly the δ^18^O values due to the lower slope of the evaporation trend (Clark and Fritz 1997).

Therefore, in order to explain these isotopic patterns, another area needs to be identified, providing a recharge characterized by both lower δ^18^O values and lower *d*-excess. Precipitation collected in the Maggiore Mt. area fulfils the first request, as rainwater collected at P1 shows an amount-weighted average of − 7.1‰ in δ^18^O, a value that could justify the lower delta values in groundwater from the Ferrarelle basin. However, the two wells (FM1 and FM2, red squares in Fig. 7) and the six springs tapping groundwater in this carbonate formation (red triangles in Fig. 7) show an isotopic composition of about − 6‰ and − 6.5‰, respectively. Therefore, the recharge by the Maggiore Mt. groundwater, if present, cannot be distinguished from that by precipitation over the Riardo basin, nor fully justify the observed low delta values. On the other hand, the three springs collected in the Matese Mts. located NE of the Riardo basin (Gallo Matese, Grassano and Venafro), show lower isotope ratios, and occasionally also a lower *d*-excess (Tab. S2).

The *d*-excess is mostly determined by the humidity conditions at primary evaporation from sea water (Clark and Fritz 1997). At the global scale, its average value is approximately 10‰ (i.e., that of the GMWL), but it increases for evaporation at lower humidity conditions such as those occurring in the Mediterranean, up to + 22‰ (Gat and Carmi 1970). Precipitation over the Apennine range may receive a greater contribution of precipitation condensing water vapour of more continental (i.e., from the North-East) or Atlantic origin, characterized by lower *d*-excess (Brenčič et al. 2015). These two sources of water vapour are more present in winter (Bottyán et al. 2017). On the other hand, precipitation in the Riardo and Roccamonfina areas is dominantly originated by humid air masses coming from the Tyrrhenian sea (Western Mediterranean), and is characterized by a higher *d*-excess (Gat and Carmi 1970). This vapour source region reaches its maximum contribution in autumn and spring (Bottyán et al. 2017), and, due to higher condensation temperature, precipitation is also characterized by higher δ^18^O values. This difference in the isotopic composition of precipitation over the Italian peninsula has been recently summarized and discussed by Giustini et al. (2016), who evidenced lower isotope ratios in precipitation on the Adriatic with respect to the Tyrrhenian side of the Apennines. In addition, numerous studies (Longobardi et al. 2016 and references therein) have evidenced a reduction of precipitation amount during winter, particularly consistent over the Tyrrhenian side of Southern Italy, and an increase during summer. Finally, similar seasonal patterns are recorded in precipitation from Zagreb (Croatia) in the last 40 years, leading to an overall increasing trend of *d*-excess (Bronić et al. 2020).

All these observations lead us to hypothesise that in the monitored productive wells, the increase in δ^2^H and in *d*-excess could reflect two main processes. The first is a general change in precipitation patterns, with reduced amounts during winter (characterized by lower delta values and lower *d*-excess) and increased amounts in warmer periods (characterized by higher delta values and higher *d*-excess). The second process involves a greater proportion of recharge from the Riardo basin, fed by precipitation originated in the Mediterranean area. This recharge would become dominant with respect to that from other sources, likely represented by the surrounding carbonate massifs (e.g., Matese Mts.) (Fig. 2), also fed by precipitation coming from the NE or the W (Brenčič et al. 2015). An extra basin recharge from the NE would be in agreement with the regional groundwater westward flow indicated by Minissale (2004) for carbonate aquifers in Central and Southern Italy (purple line in Fig. 7).

### Aquifer identification

According to Cuoco et al. (2020), most of the chemical variables measured in groundwater from the study area derive from a two-component mixing process, where the proposed endmembers are a deep, highly mineralized, CO_2_-rich water hosted in the carbonate basement, and a shallow, lower mineralized water hosted in the volcanic successions of the Ferrarelle Plain. However, as already mentioned, the volcanic and the carbonate aquifer are not separated, and, even for high degrees of water–rock interaction, the contribution from the volcanic aquifer can be detected, as indicated by the Ca/K ratio. Concerning trace elements, B is derived from the volcanic rocks, as indicated by its strong correlation with Li, whereas Sr also partially derives from carbonate hydrolysis promoted by the deep CO_2_ input, as suggested by the change in the Sr/Ca ratio with increasing mineralization (from 3.3*10^–3^ up to 2.5*10^–3^ weight ratio, Cuoco et al. 2010). Based on these premises, we considered the opportunity to use the B and Sr isotope ratios to trace the groundwater circuits.

Concerning boron, marine carbonates and volcanic rocks strongly differ in concentration and isotopic composition. Carbonates range between + 8 and + 26.2‰ (Ishikawa and Nakamura 1993), and more frequently around + 22.1 ± 3‰ (Hemming and Hanson 1992). Accordingly, groundwater from carbonate aquifers has a concentration of 10–90 µg/L and an isotopic composition comprised between + 18 and + 44‰ (Panagopoulos 2009), although the more enriched values reflect the isotopic composition of seawater (δ^11^B =  + 40‰). Groundwater circulating in volcanic rocks may have higher concentrations but is characterized by lower isotope ratios. For example, Pennisi et al. (2000) report concentrations in the range 230–550 µg/L, with isotopic compositions around − 5‰ in the aquifers of the Mt. Etna Volcano (Sicily, Southern Italy). Finally, clays, which could modify the original B isotope signature of marine carbonates and volcanic rocks by adsorption or desorption processes, are present as weathering products of ignimbrite deposits: these are expected to display a low δ^11^B value. Clays could also be present in flysch of marine origin and in alluvial deposits; however, in the Riardo basin these formations have a very limited thickness (Fig. 1).

**Fig. 9:**
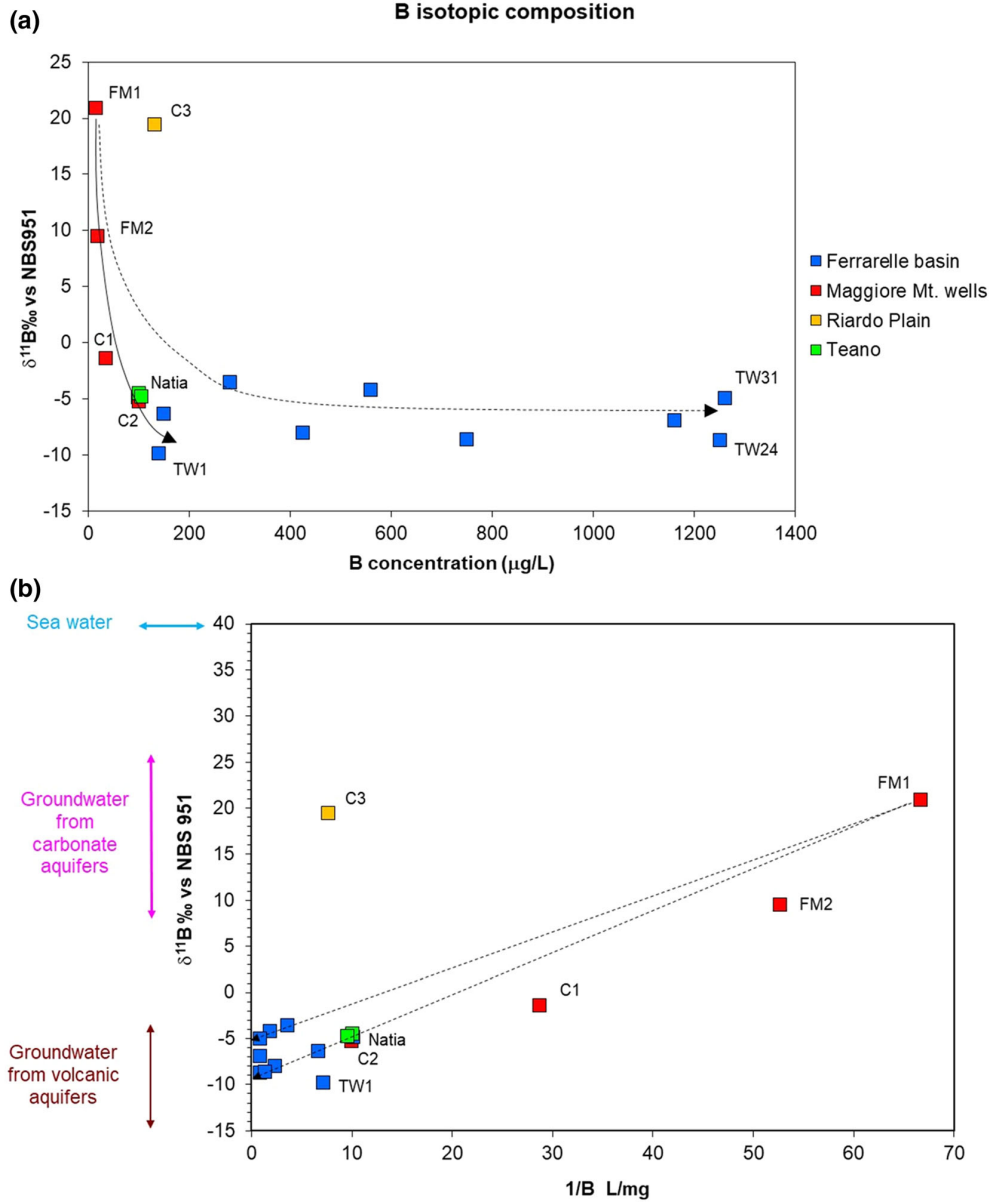
**a** δ^11^B versus B concentration; **b** δ^11^B versus 1/B. Isotopic ranges for carbonate and volcanic rocks from the literature (Ishikawa and Nakamura 1993; Paone 2008; Tonarini et al. 2004)

The relationship between the B isotope composition and concentration is reported in Fig. 9a. An overall B isotope ratio decrease with increasing concentration is observed: high values and low concentrations are displayed by samples from the Maggiore Mt. aquifer and from carbonate aquifer underneath the Riardo Plain (C3). Samples representative of the volcanic aquifer in the Ferrarelle basin (Natia) show slightly higher concentrations and isotopic compositions around − 5‰. A similar isotopic composition characterises the samples from the carbonate horst near Teano town (T1 and T2). All the other samples of the Ferrarelle basin show much higher concentrations and even lower δ^11^B values, regardless the aquifer of provenance.

In the plot of the isotopic composition versus 1/B (Fig. 9b), the compositional ranges expected for B isotopes are displayed, and the possible mixing between endmembers is represented with dashed lines. Two trends seem to be present, all compatible with the weathering of volcanic rocks, but with different isotopic compositions (about − 5‰ and − 8‰). These two values could correspond to two different types of lava of the Roccamonfina Volcano (Paone 2008): KS lava (δ^11^B = − 4.67‰), and HKS lava (δ^11^B = − 7.49‰). Even lower values (δ^11^B < − 8‰) characterise the volcanic products of the Campanian magmatic province (Tonarini et al. 2004).

In a volcanic area, the presence of B-rich fluids cannot be excluded. These could dissolve in groundwater, forming B(OH)_3_ and leading to even lower isotope ratios, such as those found in the Ischia island (δ^11^B ≈− 11‰) by Morell et al. (2008). However, the gaseous phase at Riardo is mostly constituted by CO_2_, and volatiles typical of magmatic fluids are lacking. The He isotope ratio (R/Ra = 1.89) points to a mantle origin for this gas (Cuoco et al. 2017), and no correlation is observed between the B content and the He isotope ratio, supporting the statement that B cannot be associated with it. Finally, the isotopic decrease in groundwater is also observed in samples from the Maggiore Mt. aquifer (C1 and C2) and from the Teano horst (T1 and T2), both tapping carbonate aquifers unaffected by the deep CO_2_ input. However, in the first case, thin deposits of Campanian ignimbrite outcrop in the area; in the second case, a lateral inflow of groundwater from the Roccamonfina structure could occur, as evidenced by the continuity of the piezometric map (Fig. 3).

Therefore, results confirm that the B isotope composition is dominated by water–rock interaction processes and can be explained considering the heterogeneities that are present in the volcanic aquifer, while in the Ferrarelle basin the isotopic signature of marine carbonates seems completely obliterated. To confirm this hypothesis, two samples of carbonate and volcanic rock from drill cuttings were analyzed for the δ^11^B ratio. Results indicate a value of − 7.46 ± 0.46‰ for the volcanic rock, fully in line with the expectations, and a value of − 9.43 ± 0.52‰ for the carbonate rock, even lower than the volcanic endmember.

The reasons for the low isotope ratio measured in the carbonate rock are unknown and out of the scope of this work. Nevertheless, it could be due to processes that occurred during the emplacement of the Roccamonfina volcanic system. Indeed, during hydrothermal weathering, clay minerals preferentially retain the light isotope and hydrothermal fluids are enriched (Schwarcz et al. 1969). Similarly, the low isotope ratio of the carbonate rock could be due to a selective mobilization of the heavy B isotope in the fluid phase. Whatever the reason of the observed low isotope ratio of the carbonate aquifer matrix, this composition appears too similar to the isotopic composition of the volcanic aquifer and therefore prevents its use for hydrogeological purposes.

Concerning Sr isotope data, Conticelli et al. (2009) report ^87^Sr/^86^Sr ratios in the range 0.709261–0.709991 for the ultrapotassic, leucite-rich rocks of the first volcanic phase, 0.706663–0.707455 for shoshonites of the second phase, and 0.707476–0.707492 for the calcalkaline deposits of the third volcanic phase. The values measured on shoshonites are very similar to those determined by Tonarini et al. (2004) on the Campanian ignimbrite. By contrast, marine carbonates from the Upper Trias to Upper Cretaceous, such as those outcropping in the study area, should be in the range 0.7068–0.7079 (McArthur et al. 2012). Springs from carbonate aquifers in Central Italy have an average isotope composition of 0.708 (Minissale 2004 and references therein).

**Fig. 10:**
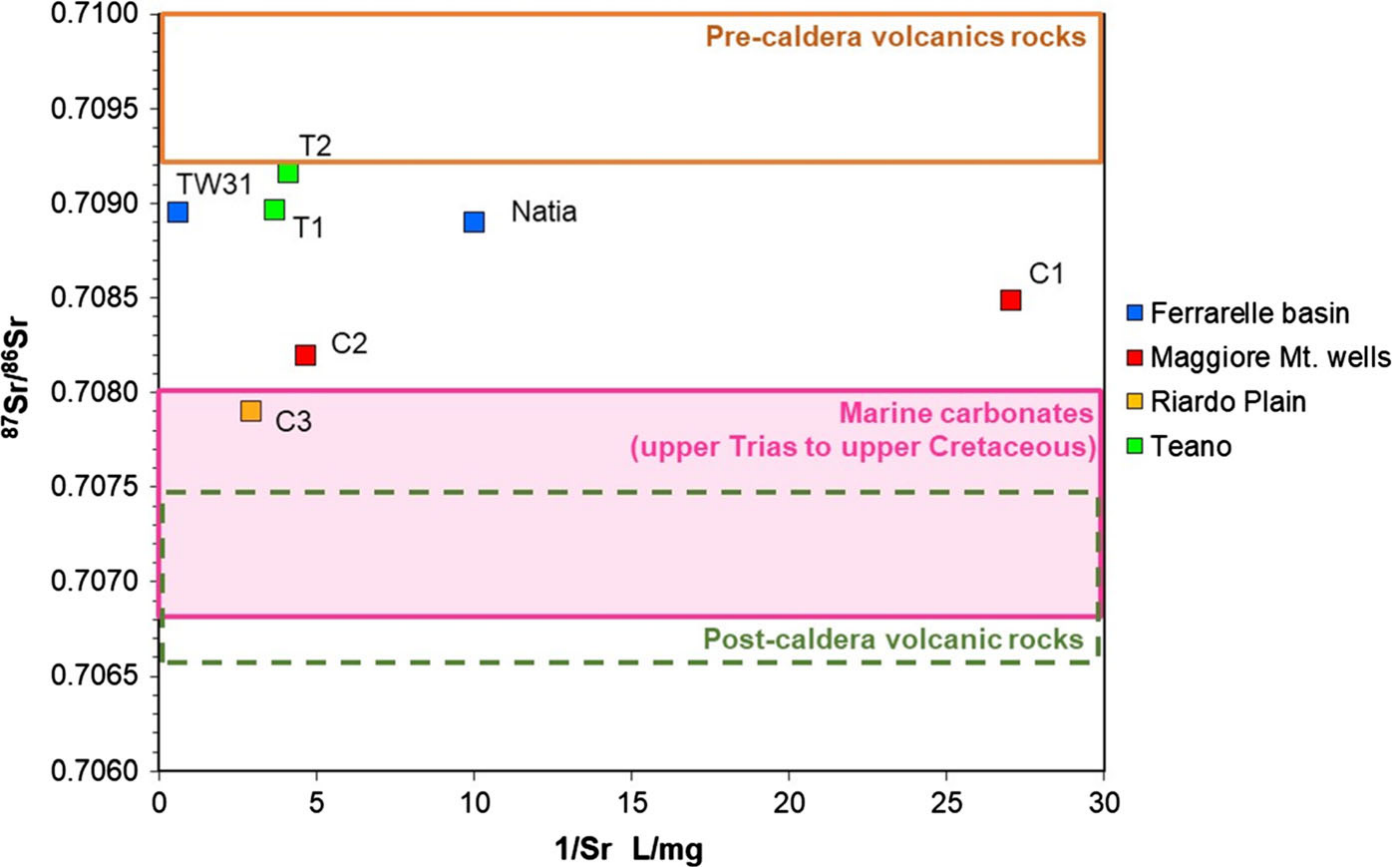
Strontium isotopic composition versus 1/Sr

The results obtained on groundwater are displayed in Fig. 10. None of the samples falls clearly in the compositional field of either rock type. Only sample C3 shows an isotopic ratio that is compatible with marine carbonates, as it did for its B isotope composition, confirming that, in the area, the aquifer matrix is little affected by post-depositional processes.

All the other samples could represent a mixture between the isotopic compositions of volcanic and carbonate rocks, although no clear mixing trends can be observed in the figure. The two samples from the Ferrarelle basin, representative of the lower mineralized volcanic (Natia) and of the higher mineralized carbonate endmember (TW31), share the same isotopic composition despite their difference in Sr concentrations. Concerning the other samples, those from the Maggiore Mt. (C1 and C2) could be affected by the presence of thin deposits of Campanian ignimbrite outcropping in the area, and those from Teano (T1 and T2) could receive a lateral inflow of groundwater from the Roccamonfina structure (Fig. 3), in agreement with their B isotope composition.

Therefore, although B and Sr concentrations proved to be good indicators of the origin of the mineralization in the Ferrarelle waters, their isotopic ratios failed to differentiate clearly groundwater circulating in the two aquifers. However, the results show that the mineralization process occurs in the carbonate aquifer of the Ferrarelle basin that shows a deeply modified isotope composition, different from the original marine signature of the surrounding carbonate reliefs.

### Groundwater dating and circulation

The interpretation of groundwater Tritium data is generally based on the Tritium content in precipitation and its variation in time due to both the anthropogenic input to the atmosphere and the radioactive decay. The nearest monitoring station is Genoa (Northern Italy), which was active from 1961 to 1995; more recent records are available for Patras in Greece (2000–2006), and Dubrovink in Croatia (2000–2003) (IAEA/WMO 2020).

Previous Tritium measurements were performed on precipitation and on groundwater samples from the Ferrarelle basin in the years 1992–1993 (Longinelli 1994). Precipitation data were in close agreement with those from the Genoa station, as shown in Fig. S2 in Supplementary Material. Groundwater data indicated the presence of Tritium concentrations ranging between 0.5 and 5 T.U. in all the considered wells, even those with higher mineralization, indicating an active recharge of the aquifers.

In this study, two samples from the Maggiore Mt. aquifer were analyzed and provided concentrations around 3 T.U. (Table 8). This value is slightly lower than present-day meteoric water for coastal Mediterranean stations (about 4.4 T.U. measured in Patras and in Dubrovinik, IAEA/WMO 2020), and indicates a current recharge for groundwater of this carbonate aquifer. Four samples from the Ferrarelle basin, that had already been analyzed in the 1993, were re-analyzed, but only one (Natia), representative of the volcanic aquifer, showed detectable levels of Tritium (0.5 T.U.; in 1993 it was 2.3 T.U). In the other samples, all the Tritium must have decayed, although TW6 displayed measurable contents in 1992–1993.

Concerning dissolved gases, several samples (e.g., Natia, TW21) show an SF_6_ excess. Since the Riardo aquifers are well protected from anthropogenic contamination, this excess is attributable to a geogenic source (Busenberg and Plummer 2000; Friedrich et al. 2013; Koh et al. 2007), as it commonly occurs in many natural environments. Some samples (e.g., Natia, Eletta) also show an excess in one or more CFCs. This type of contamination is often observed in groundwater from densely inhabited areas in Europe (Kralik et al. 2014). In this case, a geogenic source is unlikely, although studies on gaseous emissions from volcanic areas indicate that their presence cannot be excluded (Tassi et al. 2012).

Concentrations were corrected to account for temperature and pressure, based on the mean air temperature and elevation of the hypothesized recharge area. As a first step in the interpretation, a piston flow model was used to obtain a groundwater age. Then, taking into account the sampling date (Table 7), we determined the apparent infiltration year to enable the comparison of the samples among them and the correlation with the Tritium concentration data in precipitation and in groundwater. The results are reported in Table 9, together with the 1σ deviation between ages calculated with the different tracers. In most cases the 1σ deviation is comparably small, which is an indication for a good agreement between age estimates.

**Table 9:** Summary of the model ages obtained for the analyzed samples

Sample	Model age (years ± 1σ)	Infiltration year ± 1σ	Tracers used	Remarks
FM1^a^	27 ± 2	1985 ± 2	CFCs, T, SF_6_	Tracers are in good agreement
TW24	> 70	< 1942	CFCs, T	No CFCs nor T, tracers are in good agreement
TW6	58 ± 3	< 1954	CFCs, T	Presence of CFCs, absence of Tritium
F3	51 ± 1	< 1961	CFCs, T	Presence of CFCs, absence of Tritium
TW12	45–53	1966 ± 3	CFCs	Geogenic SF_6_, CFCs in good agreement
TW9	48 ± 4	1967 ± 4	CFCs	Geogenic SF_6_
TW4	48 ± 4	1967 ± 4	CFCs	Geogenic SF_6_
TW21	48 ± 4	1967 ± 4	CFCs	Geogenic SF_6_
TW26	48 ± 4	1967 ± 4	CFCs	Geogenic SF_6_
TW27	47 ± 3	1968 ± 3	CFCs	Geogenic SF_6_
P4	44 ± 10	1971 ± 10	CFCs	Geogenic SF_6_ discrepancy between age calculated with CFC-12 (53 years) and CFC-11 (34 years)
TW1	42–49	1968 ± 3.5	CFCs	Geogenic SF_6_, CFCs in good agreement
P12	35 ± 2	1980 ± 2	CFCs	Geogenic SF_6_
Natia	36 ± 1	1977 ± 1	T, CFCs	Presence of T, CFC-12 in excess, geogenic SF_6_
Natia2	28–34	1983 ± 3	CFCs	Geogenic SF_6_, CFC-12 in excess
FG	no dating	1961*	none	Gas bubbles, CFCs close to detection limit
TW30	no dating	1951*	none	Gas bubbles
Eletta	no dating	1961*	none	Gas bubbles, CFCs in excess

^a^ = well tapping the Maggiore Mt. aquifer; * = year of infiltration calculated based on the correlations of Fig. 10 (see text for explanation)

**Fig. 11:**
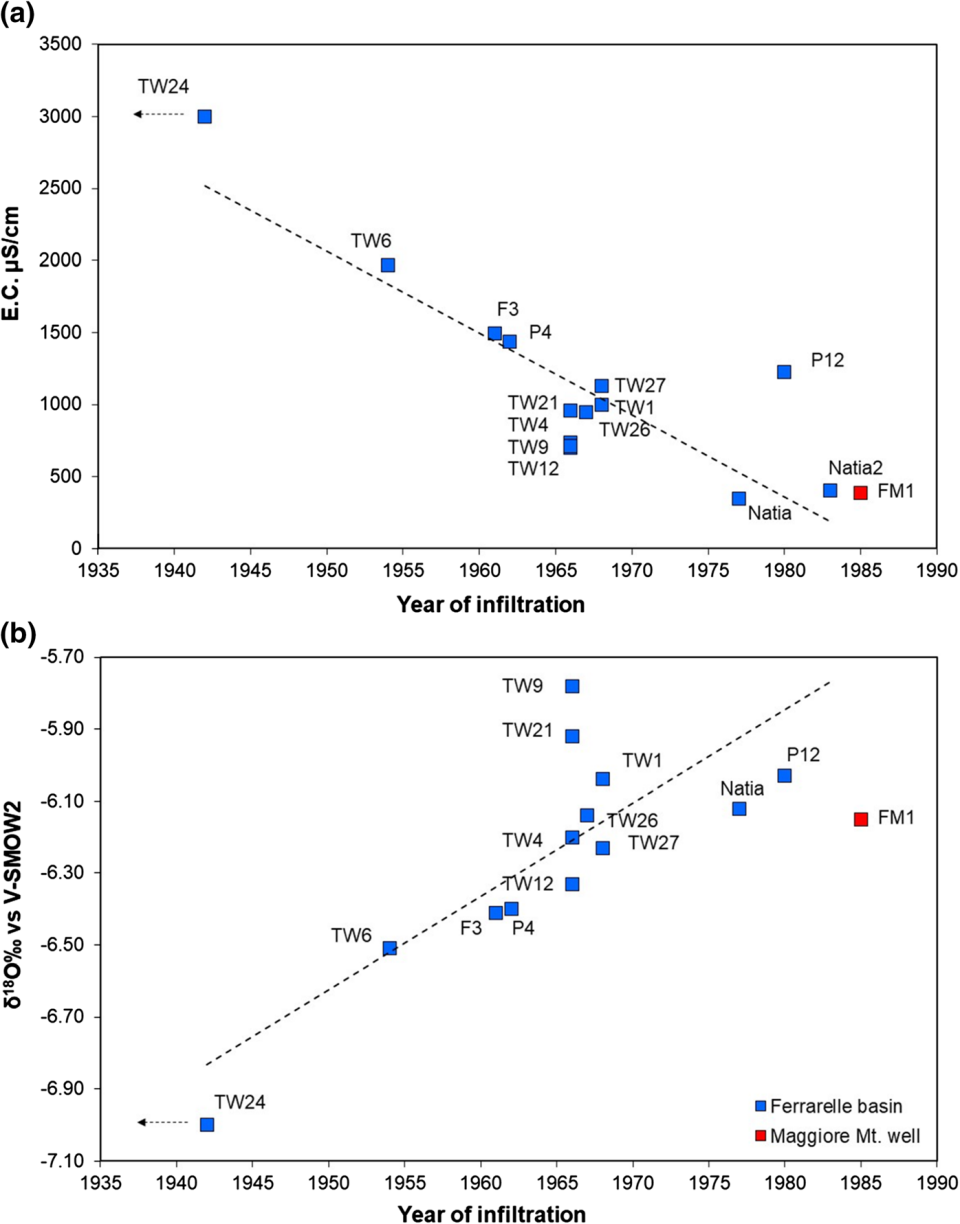
Correlation between (**a**) E.C. and (**b**) δ^18^O with the calculated infiltration year. For Natia2, no δ^18^O values are available; for P4 the older model age was considered, as better in agreement with the other data. Sample FM1 from the Maggiore Mt. aquifer was not used for the correlation

Tritium measurements were used in this study to cross validate the chronological information provided by CFCs and SF_6_. First, we used the infiltration year calculated with CFCs and the Tritium concentration in precipitation collected in the Genoa station to derive the amount of Tritium expected in recharge water infiltrated in that year; and second, we calculated the amount of residual Tritium that should have been present at the time of the analysis (2012), after decay, and compared it to the content measured in the groundwater sample (Fig. S2). The comparison is excellent for well FM1 (Fig. S2a), where the Tritium estimated after decay and that measured in groundwater perfectly agree. The situation is different for the Natia sample (Fig. S2b), that should have contained about 6.4 T.U. in 2012, and instead contained only 0.5 T.U. However, it should be noted that the Tritium content measured by Longinelli in the years 1992–1993 (Table 8) was only about 2.3 T.U., and it would be unlikely that the 2012 content would be higher than that value. The same discrepancy is shown by sample F3 (Fig. S2c), that, if infiltrated around 1961, should now contain about 4.3 T.U., but the measurement was below detection. The calculated infiltration year was then compared with E.C and with the oxygen isotope ratio in water (Fig. 11). A significant inverse correlation exists (*n* = 14; *r* = − 0.85; *p* < 0.01) with E.C., whereas a significant positive correlation is observed with δ^18^O values (*n* = 13; *r* = 0.80; *p* < 0.01).

The correlation between E.C. and the model age is easily explained, as longer residence times imply enhanced water–rock interaction and the consequent increase in mineralization. By contrast, the correlation between the isotopic composition and the model age is harder to explain. Indeed, under ideal conditions of piston flow, any given water portion would move in the aquifer with the same velocity and negligible dispersion and mixing, and water would preserve the isotopic characteristics of precipitation from the recharge area (Gonfiantini et al. 1998).

Alternatively, CFCs data could be interpreted as a binary mixture between an older, highly mineralized groundwater, recharged at higher elevations and containing no CFCs, and a lower mineralized, younger groundwater recharged in the Riardo basin. This hypothesis was tested using CFC-11 and CFC-12, because SF_6_ concentrations are often in excess and CFC-113 concentrations are very low (Table 7). In the binary plot of CFC-11 versus CFC-12 (Fig. S3 in the Supplementary Material), a mixing line is visible, having as end-members Natia (younger water, with a CFC-11 piston flow age of 36 years, consistent with that provided by CFC-113, but contaminated with CFC-12) and a CFC-free water. For the wells falling on this mixing line, calculations indicate a percent contribution of younger Natia-type water ranging from 0% (TW24) to about 15% (TW27) (Tab. S4). However, no linear correlation exists between E.C. and concentrations of CFC-12 or CFC-11, which in principle should be conservative tracers.

Uncertainties affect groundwater age determination, but also E.C. and δ^18^O, which are subject to fluctuations in time. Whatever groundwater circulation model is retained, the observed correlations of Fig. 11 suggest that the more mineralized waters are recharged at higher elevations and have longer circuits. This would then allow an indirect dating for samples such as FG and Eletta (approximate infiltration year 1961) or TW30 (1951).

**Fig. 12:**
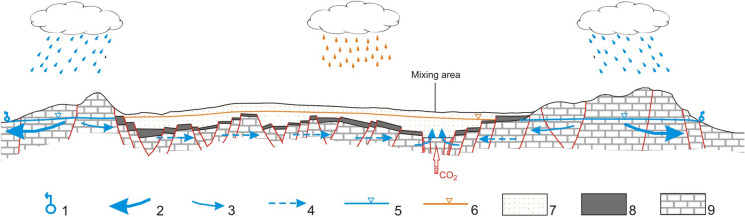
Schematic conceptual model of the Riardo Plain hydrogeological system (Viaroli et al. 2018). Not in scale. This is not a hydrogeological cross section. Legend: (1) Main carbonate springs; (2) Main discharge direction of the carbonate aquifers outside the plain; (3) Minor discharge directions in the phreatic carbonate aquifer; (4) Minor discharge directions in the confined carbonate aquifer; (5) Carbonate aquifer water level; (6) Volcanic aquifer water level; (7) Volcanic units; (8) Clays and flysch units; 9) Carbonate units. The orange and the blue rain droplets distinguish the direct recharge over the Riardo Plain and the indirect recharge over the extra-basin aquifers, respectively.

All the results discussed here support the hydrogeological conceptual model proposed by Viaroli et al. (2018) based on the groundwater budget calculation (Fig. 12). The evidence of an additional groundwater inflow recharged by precipitation over the carbonate structures located outside the Riardo basin is also supported by groundwater dating and by isotopic data. The present conceptual model agrees with the presence of a piston flow of groundwater through the sedimentary basement under the volcanic and terrigenous deposits towards the mixing area in the Riardo Plain.

## Conclusion

In this study, we investigated the groundwater circulation and mineralization processes occurring in the Ferrarelle and in the Riardo basin, using geochemical and isotopic data. Some of the findings agree with results and conclusions from previous investigations. In particular, hydrochemical data confirm the presence of two groundwater types with low mineralization, one circulating in the volcanic aquifer (e.g., Natia, of Na(Ca)-HCO_3_ type) and one circulating in the carbonate aquifers (e.g., FM1 of Ca-HCO_3_ type). The input of deep CO_2_ promotes water–rock interaction processes, leading to the formation of the naturally sparkling Ferrarelle mineral water. This highly mineralized Ca-HCO_3_ water upraises along the fractures of the Ferrarelle field and preserves the characteristics of both the volcanic and the carbonate aquifers, which locally are not separated (Fig. 12). Boron and strontium isotopes confirm that the mineralization is acquired in the Ferrarelle basin, where the isotopic signature of marine carbonates has been deeply modified by post-depositional processes of likely magmatic origin. Therefore, at the local scale, the Ferrarelle basin behaves as a homogeneous system, despite groundwater circulation being related to the presence of fractures and of gas emissions, hence strongly controlled by tectonics. However, the isotopic ratios measured in water cannot be fully justified considering solely precipitation over the hydrological basin, which includes the Roccamonfina SE flank, part of the Riardo plain and the NW sector of the Maggiore Mt., in particular for the highly mineralized end-member. This groundwater is likely recharged in the surrounding extra-basin carbonate reliefs, and proceeds from a deeper and longer circuit, as suggested by dissolved anthropogenic gases (Fig. 12). All these groundwater types circulate in confined aquifers and are pressurized. Therefore, in each of them a circulation model by piston flow is plausible. However, when reaching the Riardo basin, the deep CO_2_ flux promotes the upraise and mixing of the different fluids along the fractures, while increasing their mineral content by carbonate hydrolysis. Therefore, some mixing or interaction between groundwaters is likely to occur at this local scale.

In such a peculiar and complex hydrogeological setting, some uncertainties and open questions remain. Concerning the mineralization process, the reason of such a deep modification of the B and Sr isotope composition of carbonates in the Ferrarelle basin is still unclear, together with its lateral and in depth extent. New analyses should be performed on other samples from the basin and from the surrounding carbonate structures, with increasing distance from the Roccamonfina Volcano, under the assumption that metasomatism is related with the emplacement of the volcanic edifice. In addition, other isotopic tracers of water–rock interaction processes should be tested (e.g., Li or Mg isotopes). Regarding the circulation model, monitoring CFCs concentrations could be helpful to discriminate piston flow from binary mixing, since in the first case an increase rate of about 5% per year should be found.

The Ferrarelle Company supports research activities aimed at improving the knowledge, preservation and sustainable exploitation of groundwater resources. This has led to a large number of piezometric, chemical, physical, isotopic and scientific studies available to date. In this framework, our results are particularly relevant for mineral water exploitation for two main reasons. The first is the identification of an extra basin contribution, especially evident for the more mineralized and valuable water, which will allow to refine the hydrogeological budget of the Riardo basin; the second is the presence a recent and active recharge of the Riardo system. Both findings will contribute to a sustainable development of the resource and allow managing the Ferrarelle basin taking into account both the need for industrial development and the resource protection.

## Supplementary information

Supplementary information

## Data Availability

Not Applicable.
