# Supplementary material for: Tracing groundwater circulation in a valuable mineral water basin with geochemical and isotopic tools: the case of FERRARELLE, Riardo basin, Southern Italy
Source: Environ Geochem Health. 2021 Mar 1;44(7):1–28. doi: 10.1007/s10653-021-00845-x (PMC9177499; doi:10.1007/s10653-021-00845-x)
Supplement: Supplementary file 1 — Supplementary information [file 10653_2021_845_MOESM1_ESM.pdf]

Supplemental Material

Tracing groundwater circulation in a valuable mineral water basin with geochemical and isotopic tools: the case of *FERRARELLE*, Riardo basin, Southern Italy

Elisa Sacchi[1], Emilio Cuoco[2], Harald Oster[3], Vittorio Paolucci[4], Dario Tedesco[2], Stefano Viaroli[5*]

[1] Department of Earth and Environmental Sciences, University of Pavia, Via Ferrata 9, Pavia, Italy
[2] Department of Environmental, Biological and Pharmaceutical Sciences and Technologies, University of Campania "L. Vanvitelli", Via Vivaldi 43, 81100 Caserta, Italy
[3] Spurenstofflabor, Wachenheim, Germany
[4] Ferrarelle S.p.A., Contrada Ferrarelle, Riardo, Italy
[5] Sciences Department, University of Roma Tre, Largo S. L. Murialdo 1, 00145 Rome, Italy

*corresponding author stefano.viaroli@uniroma3.it

Table S1 Isotopic composition and rainwater amount collected at the precipitation monitoring stations P1 and P2

Station	Elevation	Monitoring period	δ18O	δ2H	d-excess	Rain		Total rain	Amount weighted	
	m a.s.l.		*‰*	*‰*	*‰*	*mm*	*L*	*L*	*δ18O‰*	*δ2H‰*
P1	525	20/03/14 - 15/04/14	-7.39	-44.2	14.97	64	2	51.6	-7.14	-42.4
		15/04/14 - 27/05/14	-6.14	-35.5	13.61	143	4.5			
		27/05/14 - 25/07/14	-5.13	-29.9	11.15	201	6.3			
		25/07/14 - 19/09/14	-5.04	-26.5	13.85	195	6.1			
		19/09/14 - 12/12/14	-7.48	-41.9	17.97	124	3.9			
		12/12/14 - 12/02/15	-9.25	-56.0	17.98	318	10			
		12/02/15 - 21/04/15	-9.14	-56.7	16.40	226	7.1			
		21/04/15 - 12/10/15	-6.21	-39.1	10.60	175	5.5			
		12/10/15 - 18/02/16	-6.41	-37.8	13.45	108	3.4			
		18/02/16 - 07/04/16	-7.38	-43.1	15.95	89	2.8			
P2	150	20/03/14 - 15/04/14	-6.9	-42.3	12.93	80	2.5	57.4	-6.40	-38.6
		15/04/14 - 27/05/14	-5.13	-29.9	11.15	99	3.1			
		27/05/14 - 25/07/14	-5.04	-26.5	13.85	159	5			
		25/07/14 - 19/09/14	-4.52	-23.7	12.48	159	5			
		19/09/14 - 12/12/14	-7.37	-43.0	15.97	70	2.2			
		12/12/14 - 12/02/15	-8.59	-53.0	15.72	318	10			
		12/02/15 - 21/04/15	-8.72	-55.6	14.19	210	6.6			
		21/04/15 - 12/10/15	-4.97	-29.9	9.83	318	10			
		12/10/15 - 18/02/16	-5.94	-36.9	10.61	150	4.7			
		18/02/16 - 07/04/16	-6.81	-41.1	13.36	80	2.5			
		07/04/16 - 13/06/16	-5.15	-30.3	10.94	105	3.3			
		13/06/16 - 20/09/16	-6.07	-40.4	8.13	79	2.5			

Table S2 Isotopic composition of the monitored springs

Sample name	δ^{18}O	δ^{2}H	d-excess	mean δ^{18}O	std δ^{18}O	mean δ^{2}H	std δ^{2}H
	‰	‰	‰	‰	‰	‰	‰
**Gallo Inf.**	-8.02	-49.3	14.85				
**Gallo Matese**	-8.05	-49.2	15.21	-8.31	0.20	-48.6	0.74
	-8.43	-48.3	19.17				
	-8.34	-47.8	18.89				
	-8.31	-47.8	18.69				
	-8.25	-48.7	17.33				
	-8.44	-48.4	19.15				
	-8.52	-49.0	19.21				
	-8.60	-49.3	19.49				
	-8.37	-49.6	17.40				
	-8.35	-48.6	18.22				
	-8.20	-49.2	16.37				
	-7.81	-46.8	15.69				
	-8.35	-48.9	17.90				
	-8.33	-48.5	18.13				
**Grassano**	-7.74	-47.4	14.59	-7.92	0.13	-46.4	0.42
	-8.09	-46.4	18.29				
	-7.97	-46.7	17.09				
	-7.97	-46.6	17.11				
	-7.71	-45.9	15.81				
	-7.95	-45.9	17.71				
	-8.06	-46.3	18.20				
	-7.96	-46.3	17.38				
	-7.80	-46.4	15.98				
	-7.72	-46.1	15.66				
	-7.83	-46.3	16.36				
	-8.07	-46.3	18.18				
	-8.04	-46.9	17.43				
	-7.91	-45.8	17.48				
**Venafro**	-7.15	-43.8	13.41	-7.33	0.17	-42.6	0.45
	-7.53	-42.9	17.39				
	-7.28	-42.7	15.55				
	-7.26	-42.4	15.69				
	-7.19	-41.9	15.65				
	-7.43	-42.4	17.01				
	-7.43	-42.6	16.83				
	-7.50	-42.5	17.50				
	-7.04	-41.6	14.70				
	-7.12	-42.6	14.35				
	-7.29	-42.8	15.57				
	-7.49	-42.4	17.48				
	-7.55	-43.4	17.02				
	-7.37	-43.0	15.96				
**Triflisco**	-6.68	-37.2	16.27	-6.63	0.09	-37.36	0.72
	-6.57	-38.0	14.59				
	-6.56	-37.1	15.35				
	-6.64	-38.3	14.86				
	-6.65	-37.6	15.65				
	-6.67	-37.7	15.74				
	-6.54	-37.0	15.36				

	-6.69	-38.1	15.39				
	-6.71	-37.1	16.59				
	-6.74	-37.9	15.98				
	-6.58	-37.2	15.45				
	-6.71	-37.1	16.58				
	-6.40	-37.6	13.59				
	-6.73	-35.3	18.50				
**Rocchetta**	-6.17	-33.7	15.67	-6.22	0.19	-34.1	0.62
	-6.19	-33.9	15.66				
	-6.32	-34.8	15.77				
	-6.38	-34.5	16.53				
	-6.29	-34.3	15.99				
	-5.79	-33.1	13.25				
	-6.41	-34.9	16.43				
	-6.20	-33.7	15.91				
**Pozzi di Croce**	-6.08	-33.6	15.02	-6.30	0.19	-34.5	0.77
	-6.38	-35.2	15.90				
	-6.44	-34.7	16.82				
**Lago verde**	-6.75	-37.5	16.53	-6.60	0.16	-37.1	0.59
	-6.33	-36.7	13.99				
	-6.74	-38.0	15.93				
	-6.50	-36.2	15.83				
	-6.55	-37.3	15.11				
	-6.58	-36.7	15.99				
	-6.52	-37.4	14.76				
	-6.83	-37.4	17.24				
**Fontana di Monte**	-6.47	-34.9	16.90	-6.19	0.25	-33.2	1.25
	-6.41	-34.0	17.20				
	-6.73	-36.2	17.69				
	-6.02	-32.7	15.47				
	-5.87	-32.4	14.56				
	-5.86	-32.1	14.72				
	-6.22	-33.5	16.24				
	-6.10	-33.0	15.82				
	-6.22	-33.0	16.75				
	-6.09	-33.0	15.68				
	-6.21	-33.7	15.98				
	-5.99	-32.0	15.94				
	-6.24	-31.6	18.33				
**Melito**	-6.43	-35.4	16.05	-6.71	0.27	-35.8	0.37
	-6.72	-35.8	17.96				
	-6.97	-36.2	19.59				

Table S3 Isotopic composition of the monitored wells. [a] Samples collected at 100 L/min pumping rate; [b] Samples collected at 500 L/min pumping rate.

Sample name	Date	$\delta^{18}O$	$\delta^{2}H$	d-excess	mean $\delta^{18}O$	std $\delta^{18}O$	mean $\delta^{2}H$	std $\delta^{2}H$
		‰	‰	‰	‰	‰	‰	‰
**C1F**	26/04/2010	-5.98	-36.6	11.25	-6.08	0.13	-36.6	0.04
	06/09/2010	-6.17	-36.6	12.71				
**Eletta**	26/04/2010	-6.29	-38.3	12.04	-6.35	0.08	-37.7	0.76
	06/09/2010	-6.41	-37.2	14.08				
**F3**	26/04/2010	-6.37	-38.4	12.56	-6.41	0.06	-37.6	1.06
	06/09/2010	-6.46	-36.9	14.78				
**FM1**	20/09/2011	-6.05	-33.7	14.73	-6.02	0.15	-32.9	0.83
	14/05/2012	-6.15	-33.0	16.23				
	05/11/2013	-5.86	-32.0	14.85				
**FM2**	20/09/2011	-6.02	-33.3	14.82	-6.10	0.07	-33.0	0.44
	14/05/2012	-6.14	-33.3	15.80				
	22/10/2013	-6.14	-32.5	16.58				
**P12**	27/04/2010	-6.30	-39.7	10.65	-6.25	0.08	-38.1	2.28
	07/09/2010	-6.19	-36.5	13.00				
**P19**	27/04/2010	-6.56	-40.1	12.42	-6.56	0.00	-38.8	1.81
	07/09/2010	-6.56	-37.5	14.93				
**P2**	27/04/2010	-6.35	-40.3	10.51	-6.44	0.14	-38.7	2.19
	07/09/2010	-6.54	-37.2	15.16				
**P24**	27/04/2010	-6.82	-41.9	12.67	-6.89	0.12	-38.1	1.26
	07/09/2010	-6.94	-39.7	15.87				
	27/09/2011	-6.94	-38.9	16.56				
	09/05/2012	-7.01	-37.6	18.43				
	13/05/2013	-6.92	-37.9	17.48				
	20/09/2013	-6.80	-37.6	16.80				
	20/05/2014	-6.97	-38.2	17.56				
	17/09/2014	-6.96	-37.1	18.55				
	21/05/2015	-7.01	-37.7	18.37				
	02/10/2015	-6.68	-37.2	16.23				
	10/05/2016	-6.96	-37.7	17.99				
	23/09/2016	-6.68	-37.1	16.34				
	23/05/2017	-6.88	-37.5	17.53				
	30/05/2019	-7.02	-37.7	18.50				
	10/09/2019	-6.73	-37.4	16.48				
**P2C**	26/04/2010	-6.01	-36.9	11.15	-6.12	0.16	-36.6	0.50
	06/09/2010	-6.24	-36.2	13.65				
**S5**	27/04/2010	-6.74	-41.2	12.74	-6.76	0.09	-37.4	1.13
	29/09/2010	-6.72	-37.9	15.88				
	13/05/2013	-6.69	-37.6	15.95				
	02/10/2013	-6.65	-36.7	16.47				
	23/09/2011	-6.74	-38.0	15.85				

	10/05/2012	-6.79	-37.5	16.87				
	20/05/2014	-6.67	-36.4	16.94				
	17/09/2014	-6.89	-37.1	18.03				
	20/05/2015	-6.88	-37.3	17.71				
	02/10/2015	-6.75	-36.3	17.67				
	11/05/2016	-6.83	-37.2	17.46				
	23/09/2016	-6.78	-37.1	17.12				
	23/05/2017	-6.93	-37.0	18.42				
	06/09/2017	-6.56	-37.0	15.45				
	27/05/2019	-6.73	-36.6	17.29				
	11/09/2019	-6.77	-36.9	17.26				
**TW1**	27/04/2010	-5.97	-36.6	11.19	-6.04	0.10	-36.1	0.65
	29/09/2010	-6.11	-35.6	13.19				
**TW24**	26/04/2010	-6.89	-42.7	12.45	-6.92	0.11	-38.1	1.37
	07/09/2010	-6.92	-39.6	15.80				
	27/09/2011	-6.95	-38.9	16.69				
	10/05/2012	-7.00	-38.0	18.05				
	13/05/2013	-7.05	-37.5	18.94				
	20/09/2013	-6.87	-37.3	17.68				
	20/05/2014	-6.99	-38.1	17.79				
	19/09/2014	-6.75	-37.7	16.32				
	20/05/2015	-7.01	-37.5	18.63				
	02/10/2015	-6.98	-37.3	18.52				
	11/05/2016	-6.88	-37.5	17.54				
	20/09/2016	-6.88	-38.0	17.00				
	23/05/2017	-7.13	-37.5	19.57				
	06/09/2017	-6.71	-37.8	15.82				
	11/09/2019	-6.92	-37.1	18.25				
	29/05/2019	-6.85	-37.4	17.41				
**TW26**	27/04/2010	-6.10	-36.6	12.16	-6.14	0.05	-36.6	0.06
	06/09/2010	-6.17	-36.5	12.81				
**TW30**	26/04/2010	-6.44	-37.8	13.68	-6.52	0.17	-36.3	1.02
	06/09/2010	-6.46	-39.0	12.71				
	23/09/2011	-6.62	-37.4	15.63				
	09/05/2012	-6.78	-36.6	17.69				
	15/05/2013	-6.37	-36.3	14.63				
	20/09/2013	-6.50	-35.8	16.25				
	20/05/2014	-6.68	-36.4	17.01				
	17/09/2014	-6.71	-36.1	17.61				
	20/05/2015	-6.71	-36.2	17.52				
	30/09/2015	-6.70	-35.8	17.79				
	13/05/2016	-6.41	-35.4	15.88				
	22/09/2016	-6.23	-35.1	14.78				
	23/05/2017	-6.41	-35.3	16.00				
	06/09/2017	-6.30	-35.7	14.67				

	27/05/2019	-6.50	-36.0	16.01				
	11/09/2019	-6.43	-35.7	15.78				
**TW31**	23/09/2011	-6.92	-38.6	16.80	-6.87	0.13	-37.6	0.42
	10/05/2012	-6.84	-37.3	17.48				
	13/05/2013	-6.90	-38.1	17.12				
	02/10/2013	-6.75	-37.5	16.51				
	22/05/2014	-6.95	-37.9	17.68				
	22/09/2014	-6.79	-37.1	17.16				
	26/05/2015	-6.96	-37.9	17.79				
	13/10/2015	-6.94	-37.4	18.16				
	16/05/2016	-6.77	-37.1	17.08				
	27/09/2016	-6.82	-37.8	16.80				
	18/05/2017	-6.84	-37.4	17.31				
	07/09/2017	-6.62	-37.4	15.56				
	28/05/2019	-7.18	-37.8	19.59				
	12/09/2019	-6.93	-37.2	18.22				
**TW4**	26/04/2010	-6.20	-37.3	12.35	-6.20	0.00	-36.3	1.34
	06/09/2010	-6.20	-35.4	14.24				
**TW6**	27/04/2010	-6.48	-38.2	13.60	-6.44	0.16	-35.8	0.96
	06/09/2010	-6.59	-38.0	14.68				
	05/04/2011	-6.20	-35.3	14.30				
	11/04/2011	-6.24	-36.1	13.80				
	23/09/2011	-6.47	-36.4	15.31				
	09/05/2012	-6.51	-35.9	16.22				
	22/05/2014	-6.30	-35.1	15.38				
	19/09/2014	-6.57	-35.4	17.14				
	20/05/2015	-6.53	-35.7	16.53				
	23/09/2015	-6.89	-36.9	18.26				
	13/05/2016	-6.37	-35.0	16.00				
	24/09/2016	-6.50	-35.8	16.16				
	23/05/2017	-6.56	-35.0	17.47				
	06/09/2017	-6.42	-35.7	15.65				
	28/05/2019	-6.42	-35.5	15.87				
	11/09/2019	-6.28	-35.0	15.22				
**TW6[a]**	15/05/2013	-6.35	-35.7	15.10				
	16/09/2013	-6.27	-34.7	15.46				
**TW6[b]**	17/05/2013	-6.52	-35.6	16.58				
	22/09/2013	-6.32	-34.9	15.73				

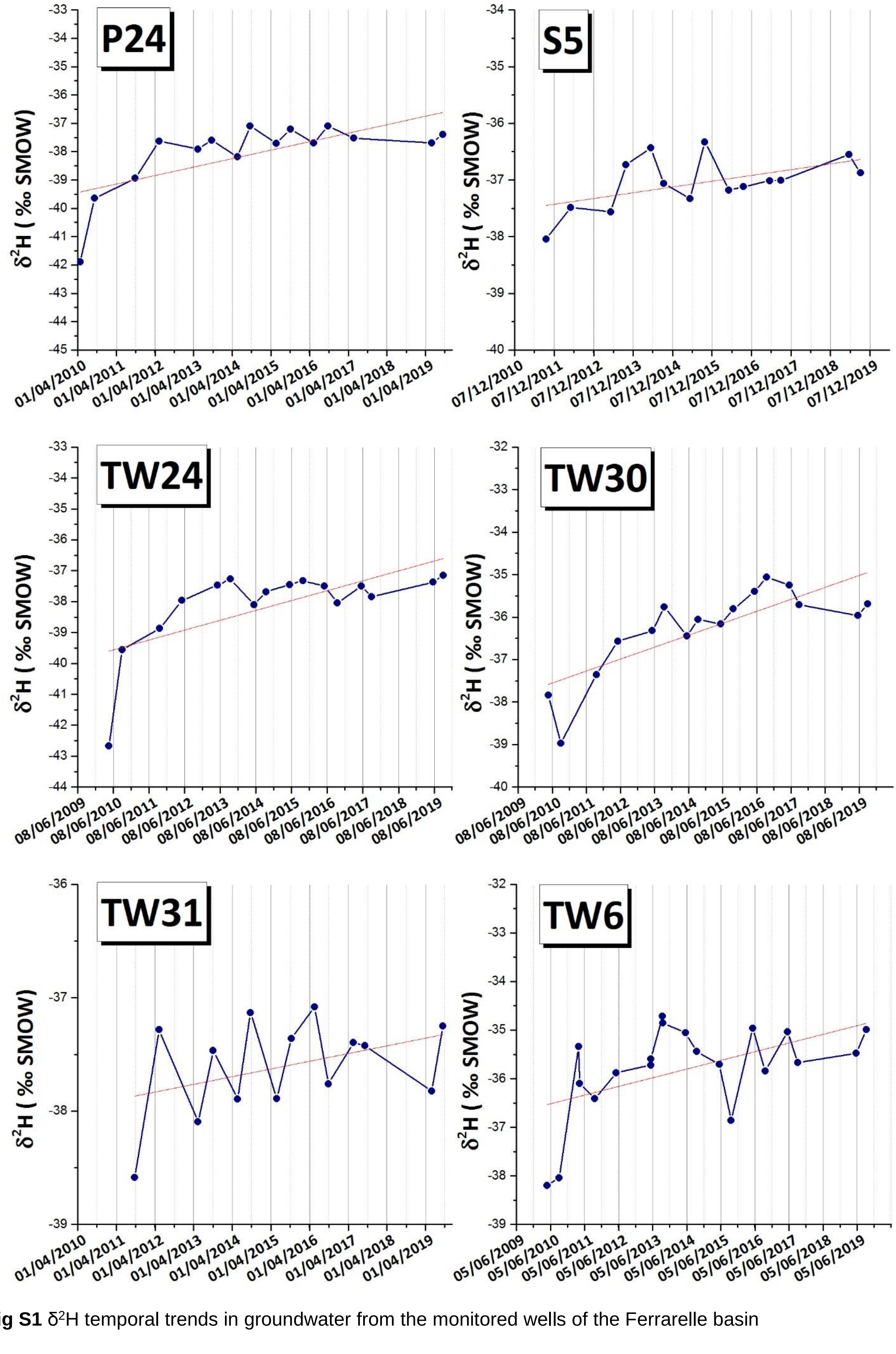

Fig S1 δ^2H temporal trends in groundwater from the monitored wells of the Ferrarelle basin

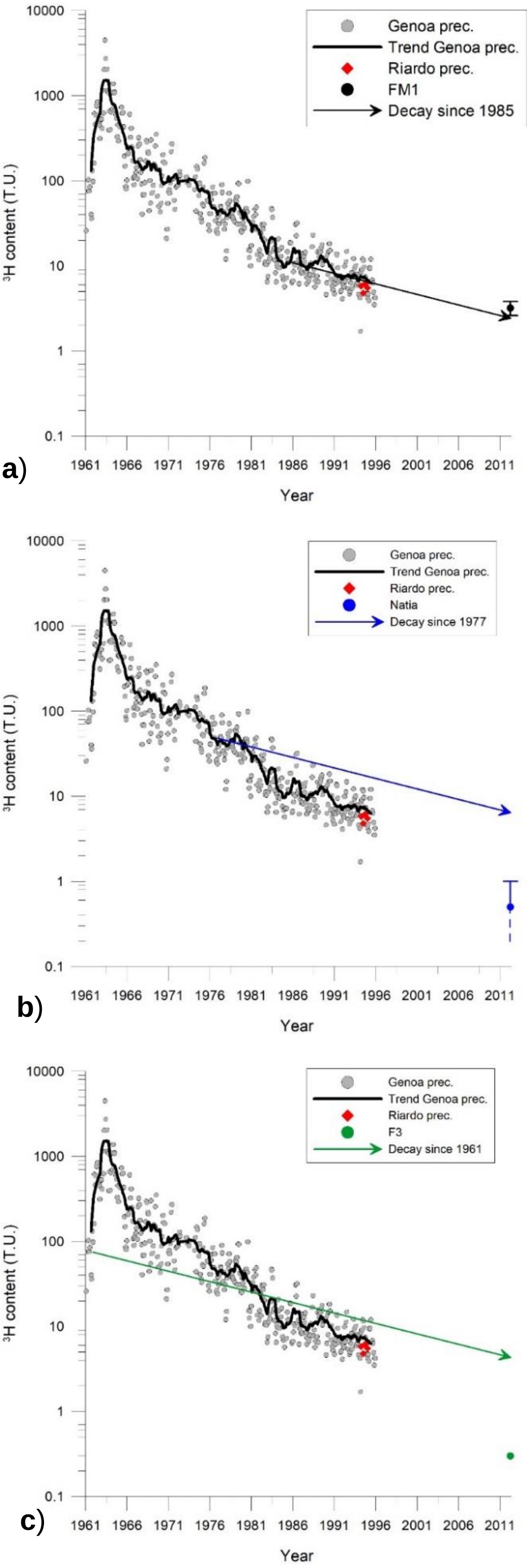

Fig. S2 Plots displaying the Tritium concentration in precipitation from the Genoa monitoring station (IAEA/WMO 2020, grey dots) and the relative trend (black curve), together with the concentration measured in precipitation from the Riardo station in 1992-1993 (red dots) (Longinelli, 1994). Tritium concentrations in groundwater from (a) FM1, (b) Natia and (c) F3, measured in 2012, compared to the concentration expected after decay, assuming that groundwater infiltrated in the year indicated by CFCs dating (arrow).

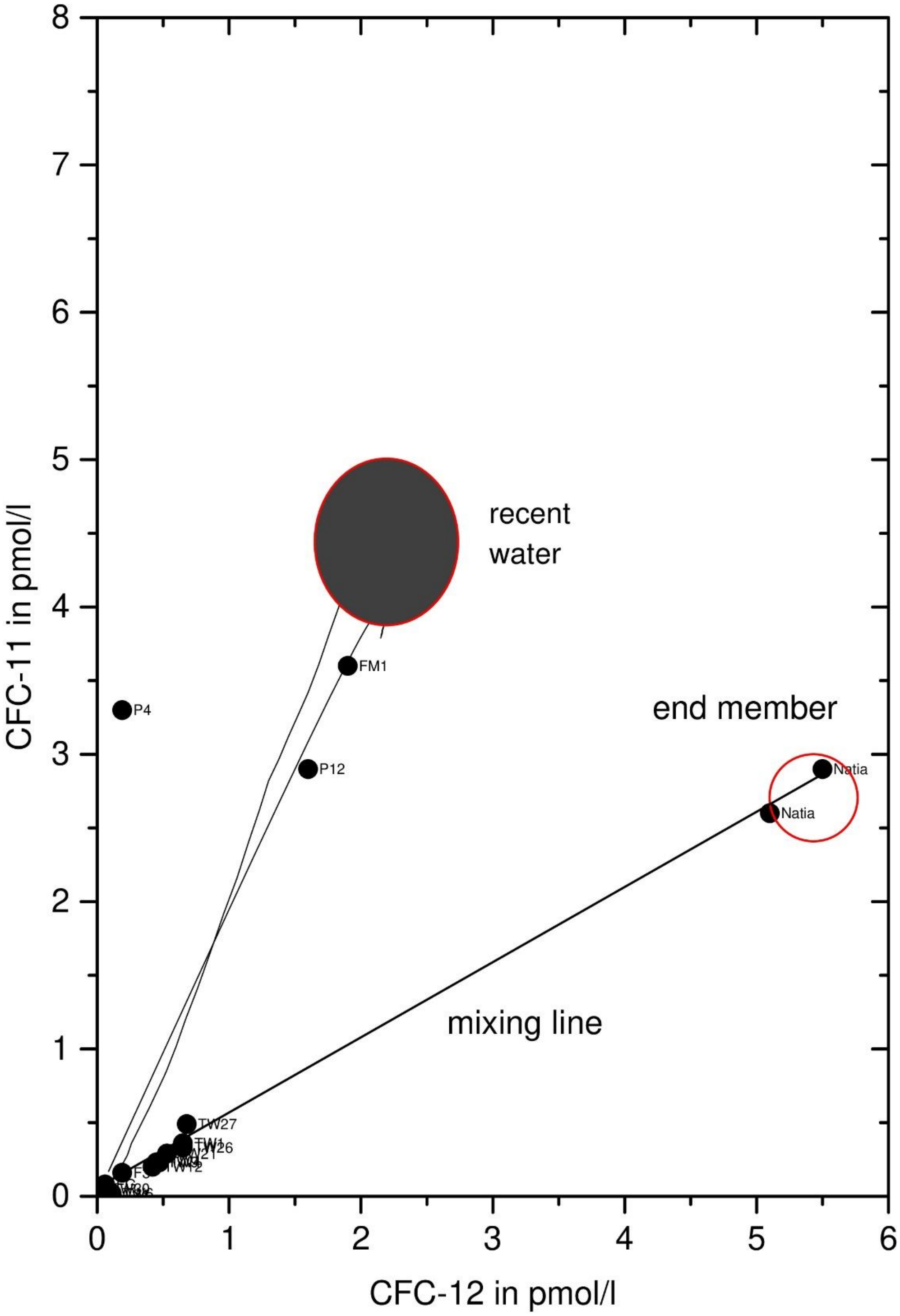

Fig. S3 Binary plot of CFC-11 versus CFC-12 concentrations in groundwater. Most of the samples fall on a mixing line having as end-members Natia (younger groundwater) and old CFC-free groundwater.

Table S4 Percent contribution of old CFC-free groundwater and of younger water (Natia-type) calculated for the samples falling on the mixing line between the two end-members (Fig. S3) using the CFC-12 and CFC-11 concentrations. The 1 sigma deviation between the two independent tracers is small for most of the samples, indicating a good agreement.

**Sample name**	**Date**	**Old comp. %** *age > 70 years*	**Young comp. %** *age 36 years*	**1 sigma %**
**TW26**	14/10/2015	88	12	1
**TW27**	14/10/2015	85	15	3
**TW12**	23/09/2014	92	8	1
**TW9**	23/09/2014	92	8	1
**TW4**	23/09/2014	91	9	1
**TW21**	22/09/2014	90	10	1
**FG**	22/09/2014	98	2	1
**TW30**	04/06/2013	99	1	1
**TW1**	04/06/2013	87	13	1
**Natia**	05/06/2013	0	104	1
**Natia**	22/05/2012	5	95	2
**F3**	23/05/2012	95	5	2
**TW6**	23/05/2012	99	1	1
**TW24**	23/05/2012	100	0	1
